# Australian wildfires cause the largest stratospheric warming since Pinatubo and extends the lifetime of the Antarctic ozone hole

**DOI:** 10.1038/s41598-022-15794-3

**Published:** 2022-08-25

**Authors:** Lilly Damany-Pearce, Ben Johnson, Alice Wells, Martin Osborne, James Allan, Claire Belcher, Andy Jones, Jim Haywood

**Affiliations:** 1 University of Exeter, Exeter, UK; 2 Met Office Hadley Centre, Exeter, UK; 3 University of Manchester, Manchester, UK

**Keywords:** Atmospheric science, Climate sciences, Climate change, Natural hazards

## Abstract

Global mean lower stratosphere temperatures rose abruptly in January 2020 reaching values not experienced since the early 1990s. Anomalously high lower stratospheric temperatures were recorded for 4 months at highly statistically significant levels. Here, we use a combination of satellite and surface-based remote sensing observations to derive a time-series of stratospheric biomass burning aerosol optical depths originating from intense SouthEastern Australian wildfires and use these aerosol optical depths in a state-of-the-art climate model. We show that the S.E. Australian wildfires are the cause of this lower stratospheric warming. We also investigate the radiatively-driven dynamical response to the observed stratospheric ozone perturbation and find a significant strengthening of the springtime Antarctic polar vortex suggesting that biomass burning aerosols play a significant role in the observed anomalous longevity of the ozone hole in 2020.

## Introduction

Sulfate aerosols originating from explosive volcanic eruptions are periodically injected into the stratosphere, where they cause a cooling of climate by scattering incident sunlight back to space (e.g.^1–3^,). Sulfate aerosols also absorb sunlight at infra-red wavelengths (e.g.^4^) causing stratospheric heating, as evident in observations subsequent to the eruptions of El Chichon and Pinatubo (e.g.^5–7^). Satellite-based instruments reveal that, with the exception of the periods influenced by El Chichon and Pinatubo, the lowermost stratosphere has cooled by around 1 K between 1979 and 1995 but has since remained approximately constant since then, primarily owing to reductions in anthropogenic emissions of ozone depleting substances (e.g.^8–10^). State-of-the-art climate models are able to represent both the long-term trend in stratospheric temperature and the sporadic heating of the stratosphere caused by the eruptions of El Chichon and Pinatubo with reasonable fidelity (e.g.^9^).

**Figure 1:**
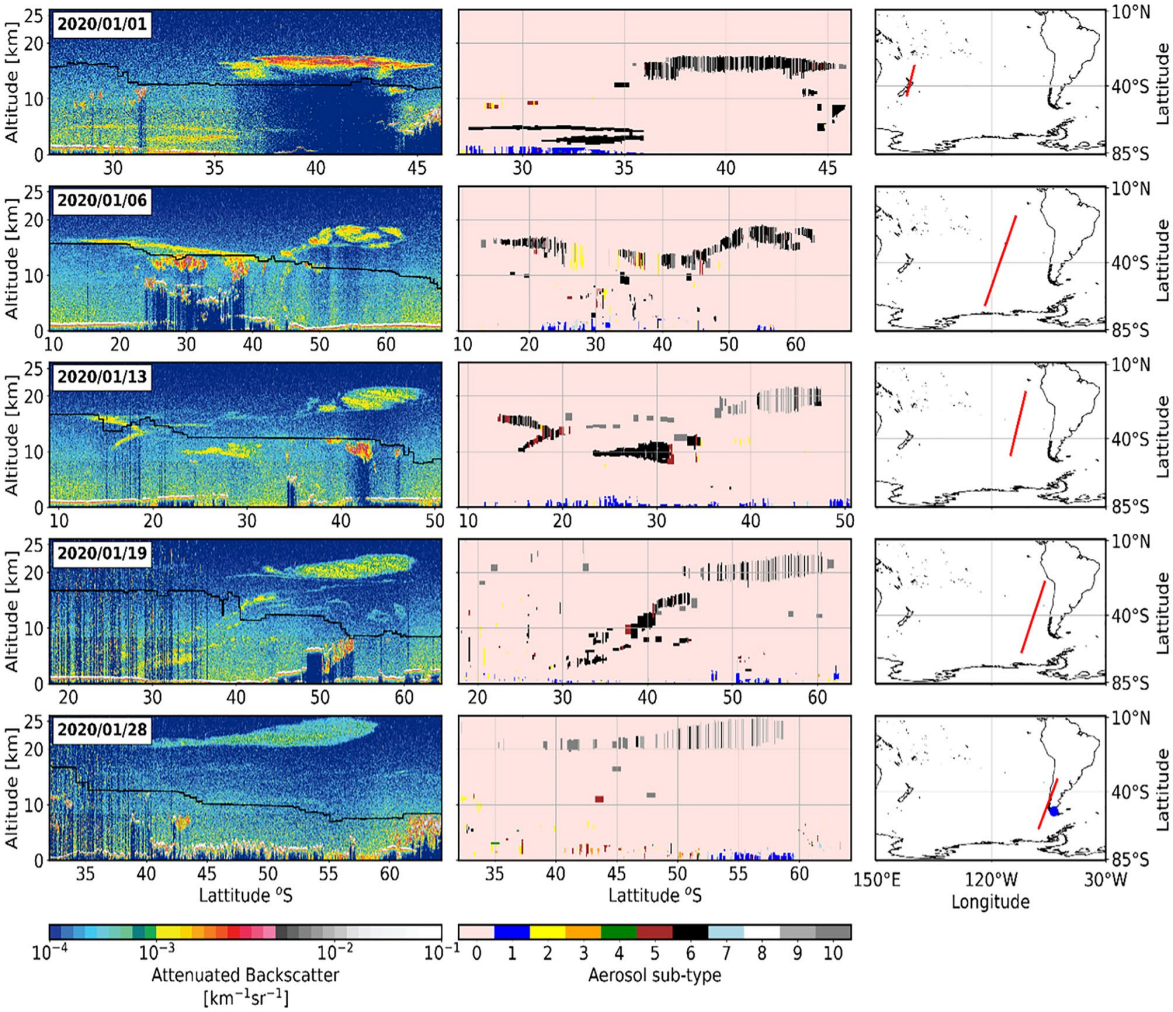
Examples of lidar observations of i) first column) the vertical distribution of the 532 nm total attenuated backscatter and the tropopause height (black line), ii) middle column) the aerosol sub-type identified by the CALIOP sensor. The third column represents the lidar footprint of the corresponding observations. The position of the Punta Arenas AERONET site is represented on the bottom right-hand plot by the blue dot in the extreme south of Chile. The maps are created using the python package, cartopy. Notable aerosol sub-types in the stratosphere include type 6-smoke, and types 9-volcanic ash and 10 – sulfate/other.

Sulfate aerosols from explosive volcanic eruptions are not the only source of stratospheric aerosols. There is increasing evidence of stratospheric intrusions of biomass burning aerosol (BBA, commonly referred to as smoke) from intense smoke infused thunderstorms, known as pyrocumulonimbus (pyroCb), generated by extreme wildfires (e.g.^11–14^). The 2019–2020 December-January–February (DJF) Australian ‘Black Summer’ wildfires were unprecedented; the scale, intensity and impacts were all unmatched in the historical record^15^ with over 5.8 million hectares burned^16^. Extreme drought caused the exceedance of critical flammability thresholds for the most prolonged period during the last 30 years ^16^ allowing the unconstrained spread of fires^15^. These intense and uncontrolled fires resulted in a multi-day pyroCb event, referred to as the Australian New Year (ANY) event^17^, resulting in millions of tonnes of smoke and associated gases being injected into the upper troposphere and lower stratosphere (UTLS)^18^. The strongest pyroCb outbreak occurred on 31 December 2019^19^, with initial injection altitudes reaching as high as 16 km^17^. The smoke plumes formed several vortices^19^, the largest of which maintained a coherent and isolated structure for over 2 months (^17^; Supplemental Fig. S1) and was eventually detected at altitudes of up to 36 km^19^. The ascent of the aerosol particles to the remarkably high altitudes is a result of the self-lofting caused by the strong solar absorption of the black carbon (BC) component of the smoke particles^20,21^, although meteorological factors also appear to play a role^22^. Figure 1 shows CALIOP-derived vertical profiles of attenuated backscatter at 532 nm and aerosol sub-type for various locations across the South Pacific as the aerosol plume evolves (the geographical evolution of the plume is shown in Supplemental Fig. S1). Over the period of a month, the aerosol plume drifted across the South Pacific and was clearly detected in the stratosphere by CALIOP as well as surface-based lidars and sun-photometers operating from the southern tip of South America^23^.

The total stratospheric aerosol optical depth (SAOD) perturbation resulting from the ANY event is on par with the strongest volcanic eruptions in the last 25 years^19^, exceeding the stratospheric aerosol intrusion produced by the previously record-breaking^13^ 2017 North American wildfires ^19,24^. It is well documented that volcanic aerosols, once in the stratosphere, can result in ozone depletion through the heterogeneous chemistry that occurs on the aerosol particles (e.g.^25,26^). Recent studies^21,27–29^ have linked the significant losses of ozone throughout the southern hemisphere, observed in 2020, to the stratospheric heterogeneous chemistry of the smoke produced by the ANY fires, through both observational and model analysis. Additionally, it has been suggested that the low levels of polar ozone may be linked to the enhanced polar vortex and low temperatures that were observed in Austral spring ^27^.

By combining satellite-based observations of BBA with surface-based remotely sensed observations of aerosol microphysical properties and utilising a state-of-the-art climate model, we add to evidence from recent studies (e.g.^21,23,24,30^,) and show beyond reasonable doubt that the substantial absorption associated with the BBA from the ANY fires caused the largest lower stratospheric warming since the eruption of Pinatubo. We also follow on from other recent work^21,27^, by incorporating satellite observations of ozone in the climate model, to further discuss the impact the ANY event had on stratospheric dynamics and the polar vortex. Our results suggest that the stratospheric cooling associated with BBA-induced ozone depletion can delay the break-up of the polar vortex, extending the longevity of the Antarctic ozone hole (e.g.^31^), which reached record levels in observations in 2020 (e.g.^32^).

## Results

### Impact on the stratospheric aerosol

**Figure 2:**
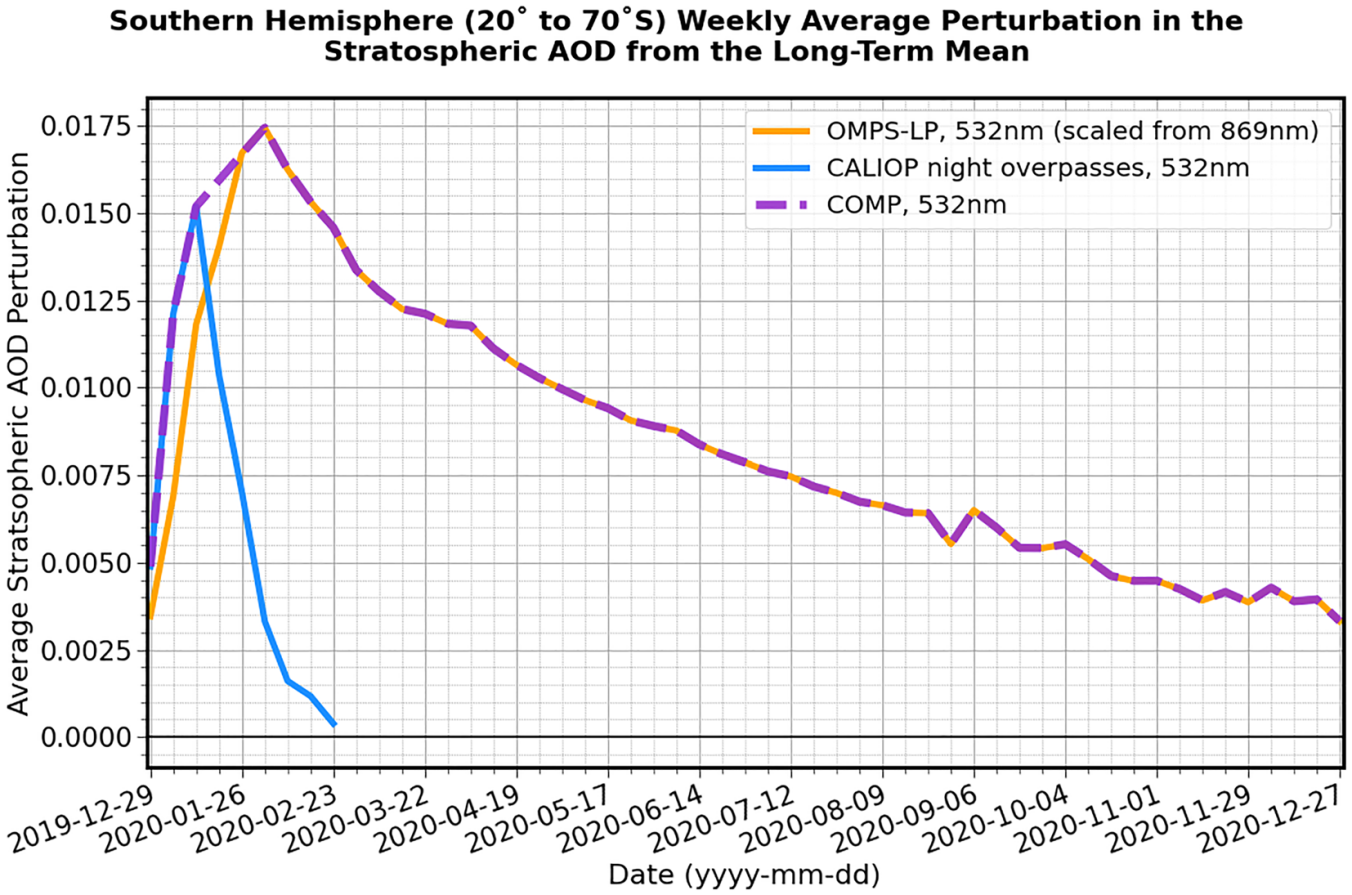
Perturbation in the SAOD (532 nm) over latitudes 20 to 70°S, as observed by CALIOP and OMPS-LP following the ANY fires. The OMPS-LP retrievals are scaled from 869 to 532 nm assuming an appropriate Ångstrom exponent. The purple dashed line is a fit that linearly interpolates between the peaks in the CALIOP and OMPS-LP retrievals, illustrating the method used to derive the composite SAOD (COMP) dataset.

We use two satellite datasets to characterise the perturbation to stratospheric aerosol in the southern hemisphere, following the ANY fires (see "methods" section for full details). The CALIOP sensor onboard the CALIPSO satellite (e.g.^33^; Fig. 1) and the Ozone Mapping Profiler Suite (OMPS) Limb-Profiler (LP) (OMPS-LP) onboard the Suomi National Polar-orbiting Partnership satellite^34,35^ are proven instruments for detecting stratospheric smoke aerosols (e.g.^14,24,36^). The perturbations to stratospheric aerosol optical depth (SAOD) above the long-term means are presented in Fig. 2 (see "M1. CALIPSO/CALIOP Aerosol Data and Quality Assurance", "M2. OMPS-LP Aerosol Data and Quality Assurance", "M3. Development of a Composite Stratospheric Aerosol Dataset" sections). It is immediately obvious that the two satellite datasets have different temporal evolutions. The CALIOP SAOD peaks at around 0.015 some two weeks after the initial pyroCb outbreak occurred. Such a delay from initiation might be expected because there were several periods of intense fires over the first few weeks of 2020, which could continue to load the stratosphere, and because aerosol injected into the upper troposphere might only enter the stratosphere subsequent to self-lofting. After this peak, the CALIOP SAOD decays rapidly because as the smoke becomes more dilute, the minimum detection thresholds for the night-time retrievals of aerosol extinction coefficient are not reached (0.012 km^−1^^37^,) leading to under-detection. As a dedicated stratospheric limb-profiler, OMPS-LP has a much lower minimum detection threshold and the SAOD reaches a maximum in early February before decaying at a much-reduced rate compared to CALIOP. Quantitative comparisons between OMPS-LP and CALIOP during the initial stage of the smoke plume suggest that, in common with other limb-instruments, OMPS-LP may fail to detect a portion of the plume that is close to the tropopause (e.g.^38,39^), although this issue appears a relatively minor concern later on in the period of this study as the aerosol reaches high altitudes. We derive a composite SAOD (COMP) data set that combines aerosol extinction from both sensors and interpolates OMPS-LP data in latitudinal bands to account for missing data during the southern hemisphere winter (see "M2. OMPS-LP Aerosol Data and Quality Assurance", "M3. Development of a Composite Stratospheric Aerosol Dataset" sections). The resulting SAOD initially follows CALIOP up to its peak and is then linearly interpolated in the ‘cross-over region’ between the peak SAODs of the two instruments before following the OMPS-LP data. We estimate a mass of BBA in the stratosphere of approximately 0.81 Tg derived from the peak of the SAOD measurements using a specific extinction at 532 nm for BBA of 3.26m^2^g^-1^ (Supplemental Section S4). This estimate is between those from previous estimates that range from 0.2 to 3.1 Tg^17–19,21^ and rivals the impact of the 2019 eruption of Raikoke which injected an estimated 0.9–1.1 Tg of SO_2_ into the lower stratosphere^40^.

**Figure 3:**
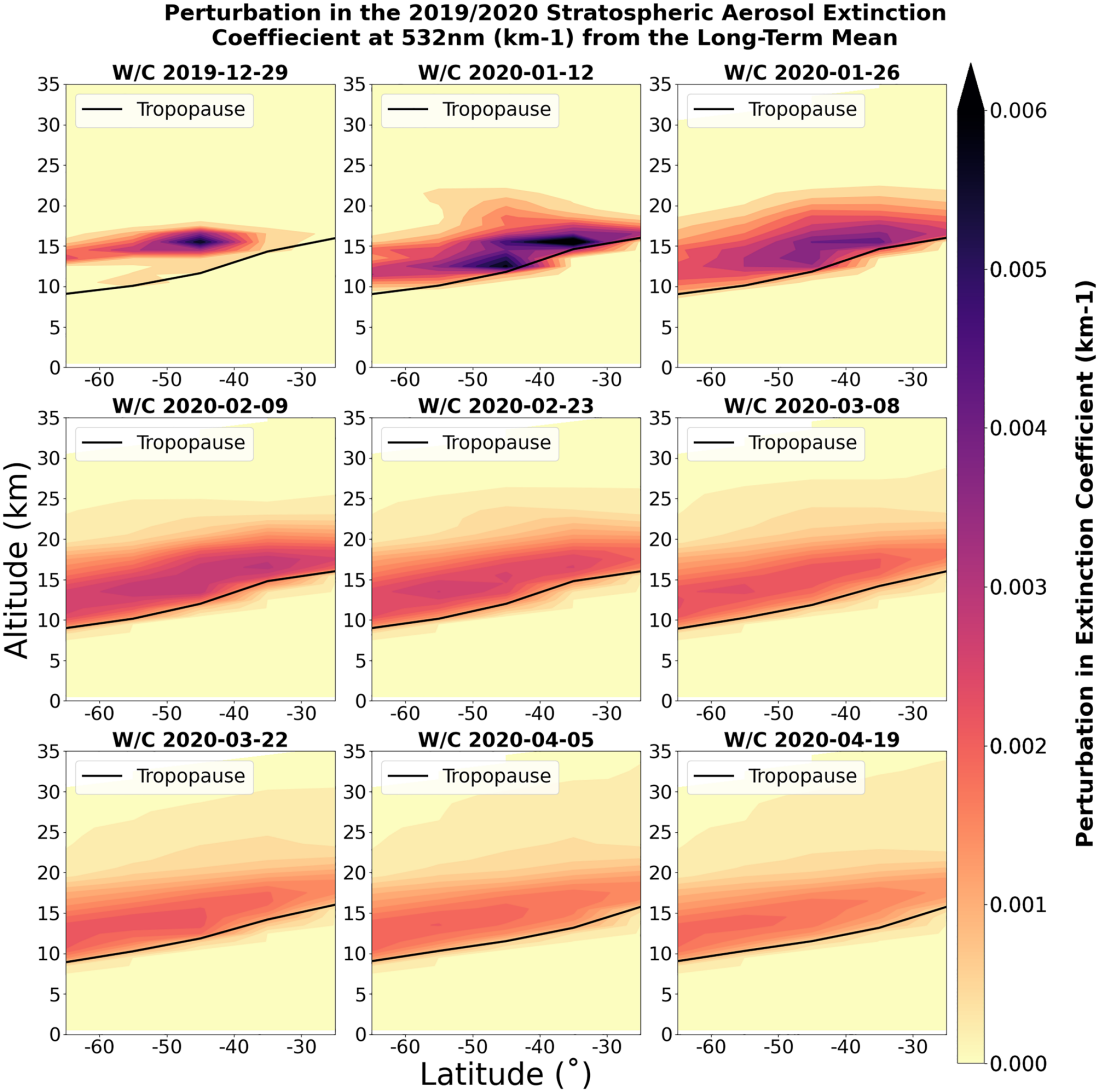
Perturbations in aerosol extinction coefficient due to the ANY fires, as a function of latitude and altitude derived by the COMP interpolated dataset. Each image shows the weekly mean distribution, for the week commencing (w/c) the date shown, for the 10-degree latitude intervals from 20 to 70˚S, and 1 km height intervals from 0 to 35 km. The black line shows the average tropopause height from the UKESM1 model climatology.

Figure 3 shows snapshots of the aerosol extinction derived from the COMP dataset at 2-week intervals to demonstrate the evolution of the smoke plume in the latitudinal-vertical plane. The first plot in the time series clearly identifies the initial injection of smoke that was observed to reach altitudes of 16 km on 31 December 2019 ^17,19^ and was transported southeast from the region of emissions creating the peak in extinction seen at 40–50°S. The south-eastward movement of the initial plume is evident from the spatial analysis of the OMPS-NM nadir viewing instrument (Supplemental Fig. S1). By the week commencing 12 January 2020, this is joined by another intense plume centered between 30 and 40°S because of further intense SE Australian fires, reaching peak values exceeding 0.006 km^-1^ in the zonal mean at altitudes of around 15 km. Subsequently, the plume spreads equatorward and dilutes significantly, but a zonal mean anomaly in aerosol extinction is still clear for the week commencing 19 April 2020 (Fig. 3) and extends throughout 2020 (Fig. 2). There is also evidence of perturbations to aerosol extinction reaching altitudes of 25–35 km, supporting the evidence shown in CALIPSO retrievals (Fig. 1) and earlier analyses^19^. Previous work,^30^, derived perturbations in aerosol extinction relative to a single month of OMPS-LP observations (December 2019) rather than relative to a long-term mean, but we note many similarities between their results and those shown here.

### Impact on the stratospheric ozone

**Figure 4:**
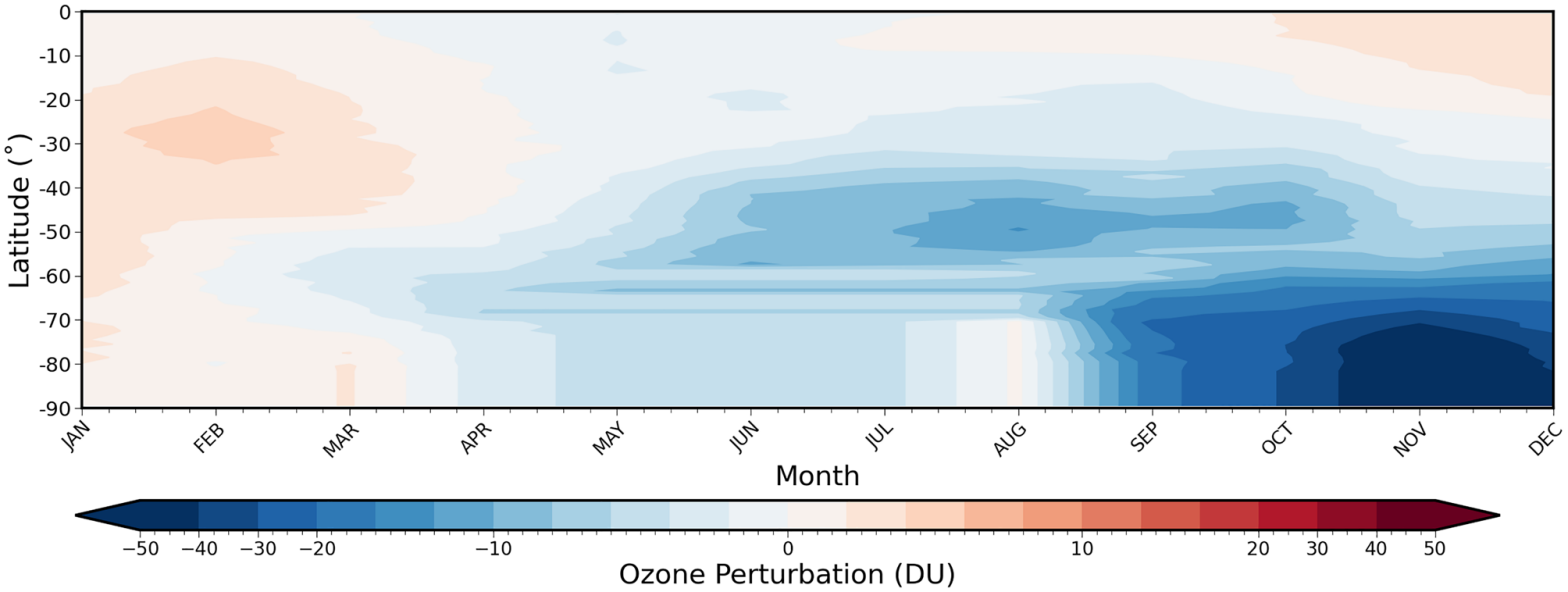
UTLS ozone anomaly (DU) over the southern hemisphere, retrieved by OMPS-LP. The ozone vertical profiles are vertically weighted using the RSS vertical weighting function (see Eq. (1), Stratospheric Temperature Anomaly Data) then integrated over altitudes 13–22 km.

Previous work^21^ has used model simulations to predict persistent ozone negative anomalies in the SH from August to December 2020, resulting from the heterogeneous chemical reactions that occur on the surface of the smoke aerosols. This impact on ozone has been further analysed through the use of both OMPS-LP and MLS satellite-based observations of stratospheric aerosol, ozone, and temperature to conclude that the observed mid-latitude and polar ozone loss was a result of the smoke produced by the 2020 ANY fires^27^. In a similar approach, we use the OMPS-LP retrieved ozone vertical profiles to consider the observed ozone anomaly over the southern hemisphere in 2020, focussing on altitudes of 13–22 km. Figure 4 shows the latitudinal structure of the 2020 stratospheric ozone anomaly calculated from the long-term mean (see also^27^ their Fig. 1B, although a different long-term mean is used here–see section "M4. OMPS-LP Ozone Data and Quality Assurance"). As with the aerosol extinction data, we account for the missing OMPS-LP data, during the southern hemisphere winter, by applying an appropriate interpolation to the data (see section, "M4. OMPS-LP Ozone Data and Quality Assurance"), with the resulting ozone anomalies shown in Fig. 4. Figure 4 shows a similar distribution of mid-latitude ozone loss to that shown in other studies^21,27,28^, although the magnitude of ozone loss shown here, 12DU, is greater than for previous model simulations, 7DU (^21^, their Fig. 4b), which account only for heterogeneous chemistry. The significant observed BBA-induced mid-latitude ozone loss has been shown to be similar to that observed after the 2015 eruption of Calbuco^27^. Figure 4 also shows the large Antarctic ozone hole, that reached near record levels in 2020. Since stratospheric ozone depletion can have significant impacts on both stratospheric temperature and dynamics, especially in the polar region^31^, we include the observed ozone anomaly from April 2020-December 2020 in our model simulations.

### Modelling the impacts of the aerosol and ozone perturbations

To estimate the impact that the perturbations have on lower stratospheric temperatures (LSTs), we apply the COMP extinction and OMPS-LP ozone perturbations to the UKESM1 climate model. UKESM1 has a facility (Easy-Aerosol) for imposing distributions of aerosol optical properties (see section "M5. The UKESM1 climate model and simulations"). To extend the extinction into full wavelength-dependent optical properties we assume an aerosol size distribution derived from AERONET observations over Punta Arenas in southern Chile which was clearly impacted by the BBA from the ANY fires (^23^, their Fig. 1). We assume the refractive indices derived from recent intensive measurements of aged biomass burning aerosol^41^. Mie scattering calculations reveal a single scattering albedo at 550 nm of 0.86, which is representative of aerosol with strong absorption characteristics which has also been inferred from co-located lidar measurements of the stratospheric smoke^23^. The absorption properties of BBA in the troposphere have been the subject of much scrutiny in recent international field campaigns (e.g.^42,43^) and depend on the combustion sources, the degree of flaming and smouldering combustion, age dependent oxidation, bleaching of organic components, and the collapse of black carbon chain structures (e.g.^42^). The optical properties associated with UTLS smoke are even more uncertain (e.g.^20^). However, a single scattering albedo of 0.86 at a wavelength of 0.55 μm agrees with lidar derived estimates of 0.8–0.9 for aged boreal UTLS smoke^23,44–46^.

We perform three separate UKESM1 model simulations each comprised of 10 pairs of simulations. Each pair includes a control simulating present-day climate (CNTL) and one of three parallel simulations starting from the same initial conditions but including either the aerosol perturbation (BBA), the ozone perturbation (O3), or both the aerosol and ozone perturbations (BBA + O3). Each pair runs for 12 months, using a unique set of initial conditions to create an ensemble of results.

### Impact on stratospheric temperature

**Figure 5:**
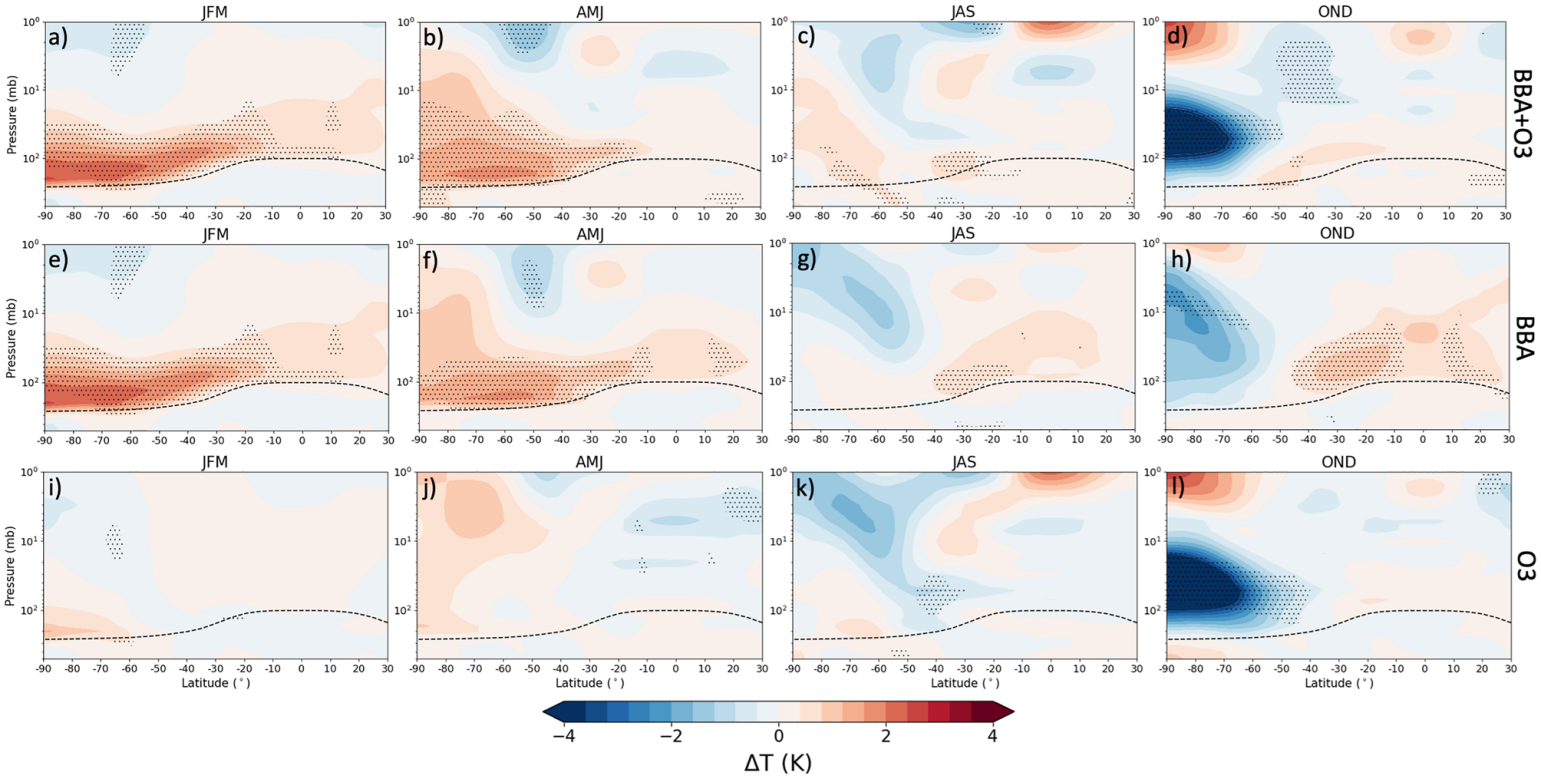
Three-monthly mean modelled perturbations to atmospheric temperature (**K**) due to the smoke aerosol forcing (**e–h**), the ozone anomaly (**i–l**), and both the smoke aerosol and ozone (**a–d**). The stippled points show the points at which the experiments were significantly different from the control simulations at a 5% significance level (calculated by a Welch’s t-test). The black dashed line shows the average tropopause height from the UKESM1 model climatology.

The radiative heating associated with the aerosol perturbation peaked at around 0.2 K/day (Supplemental Fig. S2) and the resulting perturbations to the mean stratospheric temperatures from the three model experiments are shown in Fig. 5 as zonal averages for each experiment. Statistically significant increases in LSTs (at 5% significance level), resulting from the smoke aerosols, develop across much of the southern hemisphere and persist through January–June (Figs. 5a–h). These warm anomalies peak during January-March and reach around 2.5 K at pressures of between 100 and 200 hPa (~ 12–16 km). These results are consistent with the zonal temperature anomalies produced by previous model calculations^21^ and MLS observations^27^. Both poleward and equatorward increases in LSTs, outside of the region of the imposed smoke aerosols (20–70˚S), can be seen in Fig. 5a,b,e,f. Remote temperature perturbations have been found in other model experiments that injected aerosol at 30°S to replicate the impacts of hypothetical geoengineering schemes (^47^, their Fig. 1e). This latitudinal spread of the temperature response is intrinsically linked to induced changes in stratospheric dynamics which can also influence stratospheric ozone (e.g.^47,48^). Figure 5a–d and i–l also indicates how the aerosol-induced reduction in ozone impacts the stratospheric temperatures. The impacts are particularly significant in the high latitudes during the last quarter of 2020, consistent with observations which show a particularly strong and cold polar vortex^32^. The statistically significant cooling in the polar region during October-December, with temperature anomalies stronger than − 4 K at pressures 20–100 hPa (~ 16–25 km).

To assess whether the modelled perturbation to the LST resembles reality, we examine observations from the Microwave Sounding Unit (MSU) channel 4 and Advanced Microwave Sounding Unit (AMSU) channel 9 retrievals that are sensitive to LSTs within the 13–22 km altitude band^9,10^, provided by RSS, where the model temperature perturbation is largest (Fig. 5). These instruments are chosen in preference to the Stratospheric Sounding Unit (SSU) as the minimum altitude of detection for the SSU channel 1 is around 25–35 km (pressures less than 20 hPa) which is above the altitude of the maximum temperature perturbation derived from the model. To enable a fair comparison, the model temperature data has been vertically weighted in the same way as the RSS data ("M6. Stratospheric Temperature Anomaly Data" section). We compare the LST anomalies from RSS and UKESM1 across the long-term record (1979–2020) and also more closely for 2020, focussing mainly on variations at the global scale (with near-global coverage from 83°S to 83°N), although results are also presented for sub-regions in the Supplemental Material (Fig. S4).

**Figure 6:**
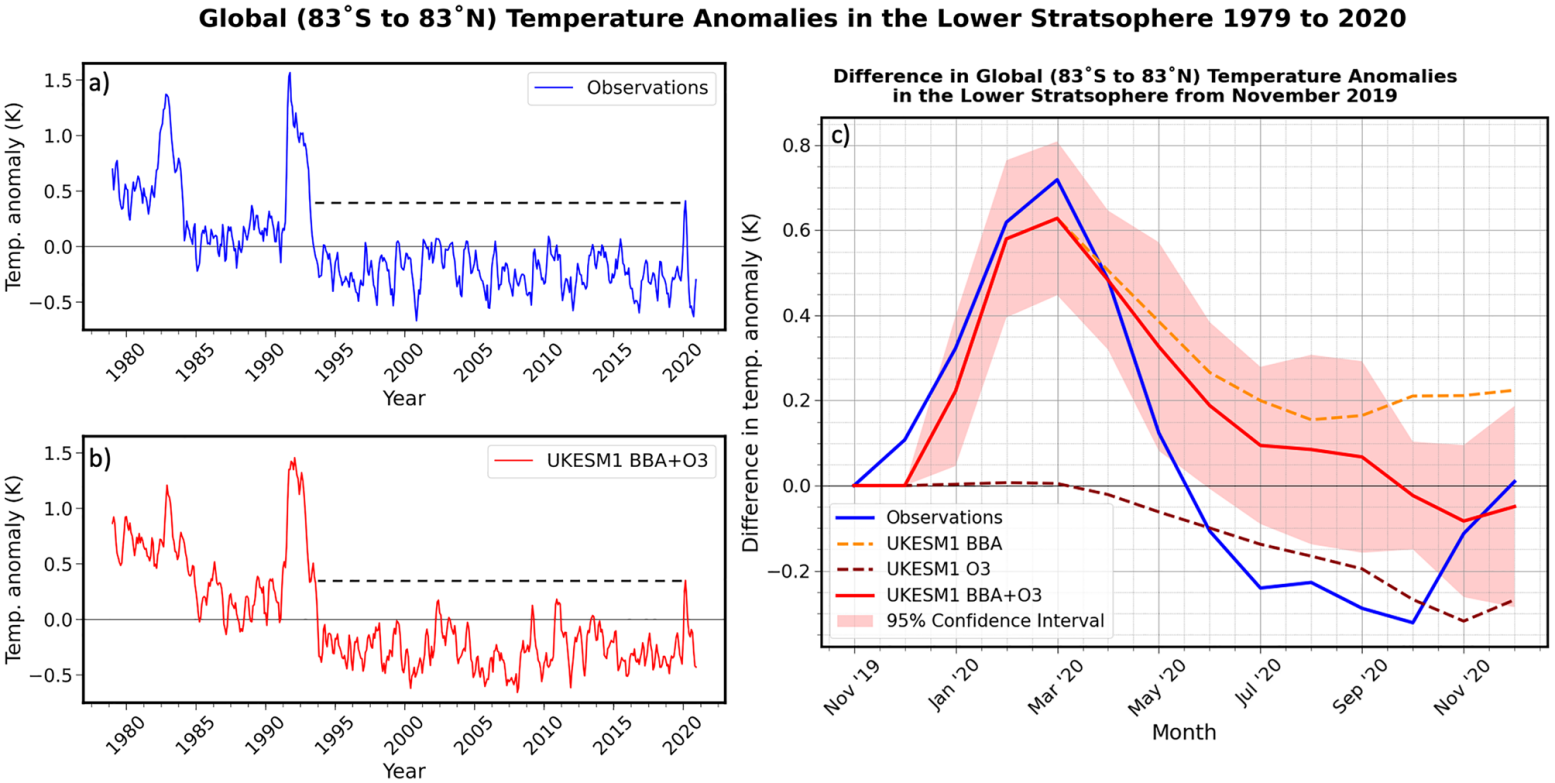
Monthly-mean lower stratospheric temperature (LST) anomalies provided by RSS data (**a**), and the mean of the 10-member ensemble of UKESM1 BBA + O3 simulations (**b**). Temperature anomalies are calculated relative to a 1979–2019 reference period, and averaged over latitudes 83˚S to 83˚N. The difference in the LST anomalies relative to November 2019 are shown in (**c**). The BBA, O3 and BBA + O3 temperature anomalies shown in (**c**) are the differences from the control simulation. In addition, the 95% confidence intervals for the BBA + O3 experiment for each month of 2020, assuming a Student’s t-distribution, are shown in c).

Figure 6a,b shows that well-known features of the observed record are well-replicated in the climate model. The long-term mean cooling trend attributed to stratospheric ozone depletion^8–10^ and the levelling off of that trend due to reduced anthropogenic emissions of ozone-depleting substances (ODS, e.g.^49,50^) are well-represented. The observed time series is interrupted by two strong warming events associated with the volcanic eruptions of El Chichon and Pinatubo which are again well-represented by the UKESM1 climate model. For the period 1995–2020 (i.e., the period not influenced by the major volcanic eruptions of El Chichon or Pinatubo), the standard deviation from the global mean temperature is 0.170 K in the observations and 0.174 K in the model, revealing that the interannual variability in the model agrees with observations. In the observations (Fig. 6a), a strong but brief warming event occurs at the beginning of 2020 and emerges as the highest spike in LST anomalies since the aftermath of the eruption of Pinatubo in 1991. This warming event is not associated with the sudden stratospheric warming event of late 2019 (e.g.^51,52^). The limited areal extent of the 2019 sudden stratospheric warming in southern hemisphere polar regions means that, while such an event is significant regionally, and even at hemispheric scales (Supplemental Fig. S4), it becomes relatively insignificant at the global scale. Figure 6b shows that, as in the observations, the sudden uptick in the modelled LST anomalies is evident as the largest since the aftermath of the Pinatubo eruption.

Figure 6c focusses on 2020 and shows the difference of the three model experiment simulations (BBA, O3, BBA + O3) from the control simulation, together with the observations. The 95% confidence interval for the BBA + O3 simulation is also shown as this is our ‘best estimate’ of the real stratospheric conditions. The LST anomaly for the BBA + O3 is statistically significant throughout February–May, with *p*-values between 0.0001 and 0.0396 for each month (assuming a Student’s t-distribution) indicating that the temperature anomalies caused by the Australian BBA are well outside those that occur naturally in the model. The temperature anomaly increased to around + 0.7 K from November 2019 to February/March 2020 in the observations and to + 0.65 K in the model simulations including BBA. The LST anomaly in the BBA experiment remained positive throughout 2020, to elucidate reasons that the BBA is so effective at warming the UTLS, we perform Mie scattering calculations averaged across the solar spectrum for both BBA and for sulfate aerosol (Supplemental, S4). Sulfate aerosol only absorbs significant solar radiation for wavelengths exceeding around 1.3microns (e.g.^4^), while BBA absorbs across all solar wavelengths. Our calculations suggest that BBA is approximately 50 times more absorbing than sulfate aerosol, and thus much more highly effective at heating the stratosphere than sulfate from e.g., volcanic eruptions. Figure 6 indicates that, although the smoke-induced ozone climatology clearly acts to decrease the LST anomaly, the smoke aerosols dominate the impact on LSTs in BBA + O3 until Austral spring, where the Antarctic ozone hole is at its greatest. The agreement between the observations and the modelled stratospheric temperatures is excellent for the January-April period when the smoke aerosol drives the stratospheric heating, but, although a global mean cooling from stratospheric ozone depletion is seen in the model, the model does not adequately simulate the magnitude of the observed cooling in the period June-October. Reasons for this discrepancy are unclear and should be the focus of future work.

### Impact on stratospheric dynamics and the SAM

**Figure 7:**
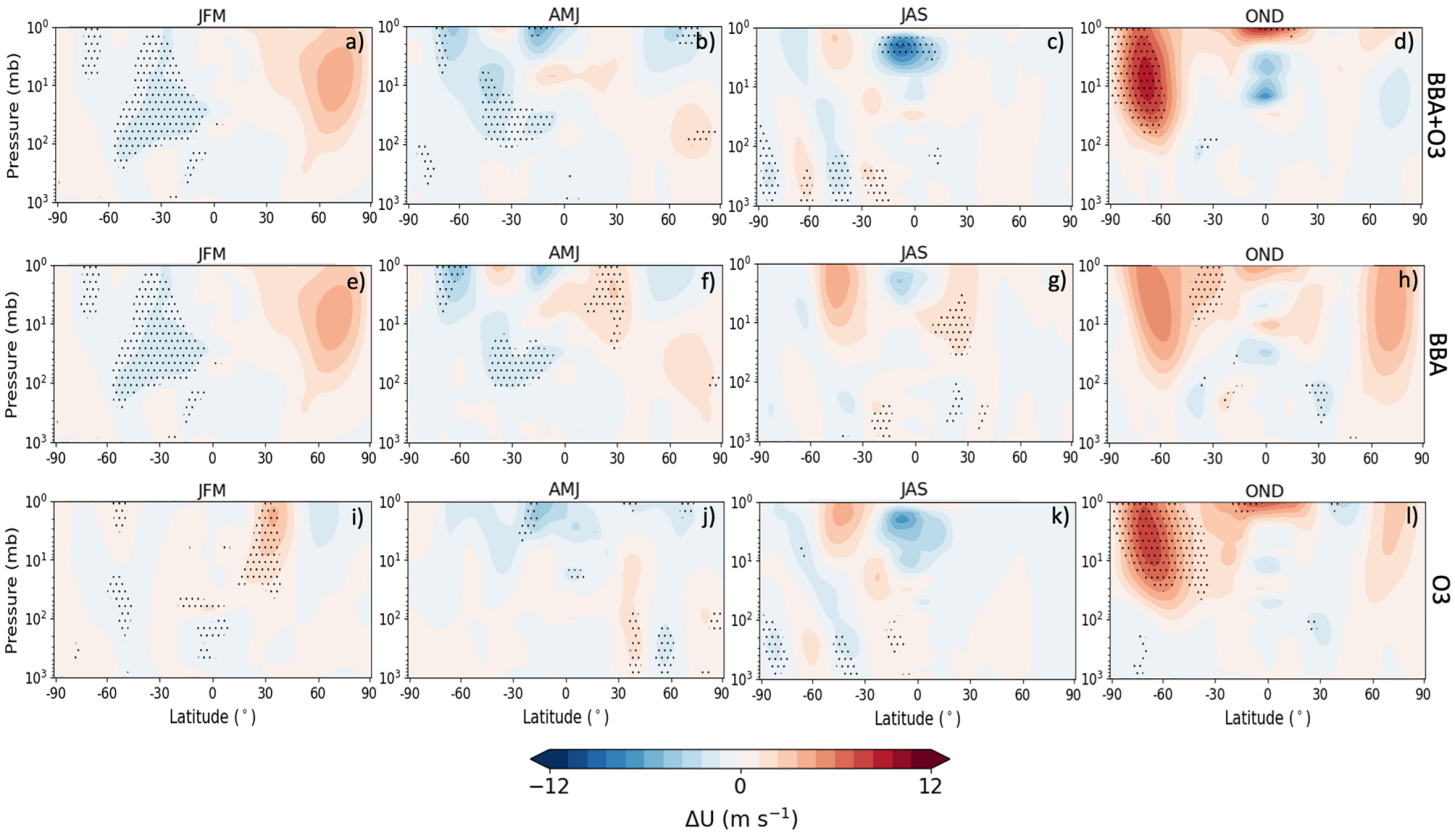
Three-monthly mean modelled perturbations the zonal mean wind (m s^−1^), over all latitudes, due to the smoke aerosol (**e–h**), the ozone anomaly (**i-l**), and both the smoke aerosol and ozone (**a–d**). The stippled points show the points at which the experiments were significantly different from the control simulations at a 5% significance level (calculated by a Welch’s t-test).

Stratospheric BBA can influence stratospheric dynamics through both the heating of the stratosphere and the response to the resulting smoke-induced ozone depletion which also changes stratospheric heating rates. Figure 7 shows the change in the zonal mean wind, for each of the three model experiments. There is a statistically significant weakening of the westerly winds by approximately 5 m s^-1^ during January-March between 20 and 50˚S, shown in Figs. 7a,e, corresponding to the time and location of the greatest stratospheric smoke anomalies (Fig. 3). The simulated changes to the zonal mean winds are less significant throughout April-September, until the period of the maximum smoke-induced ozone loss from October-December. Figures 7d,l show the significant strengthening of the zonally averaged westerly winds by about 10 m s^-1^ south of 40˚S and above 50 hPa (~ 22 km). The Antarctic polar vortex usually begins to break down towards the end of spring, allowing rapid mixing of high-ozone air across the vortex boundaries^53^. Statistically significant (at the 5% level) increases in the westerly winds in the high latitudes suggest an acceleration of the polar vortex and a subsequent delayed breakdown.

**Figure 8:**
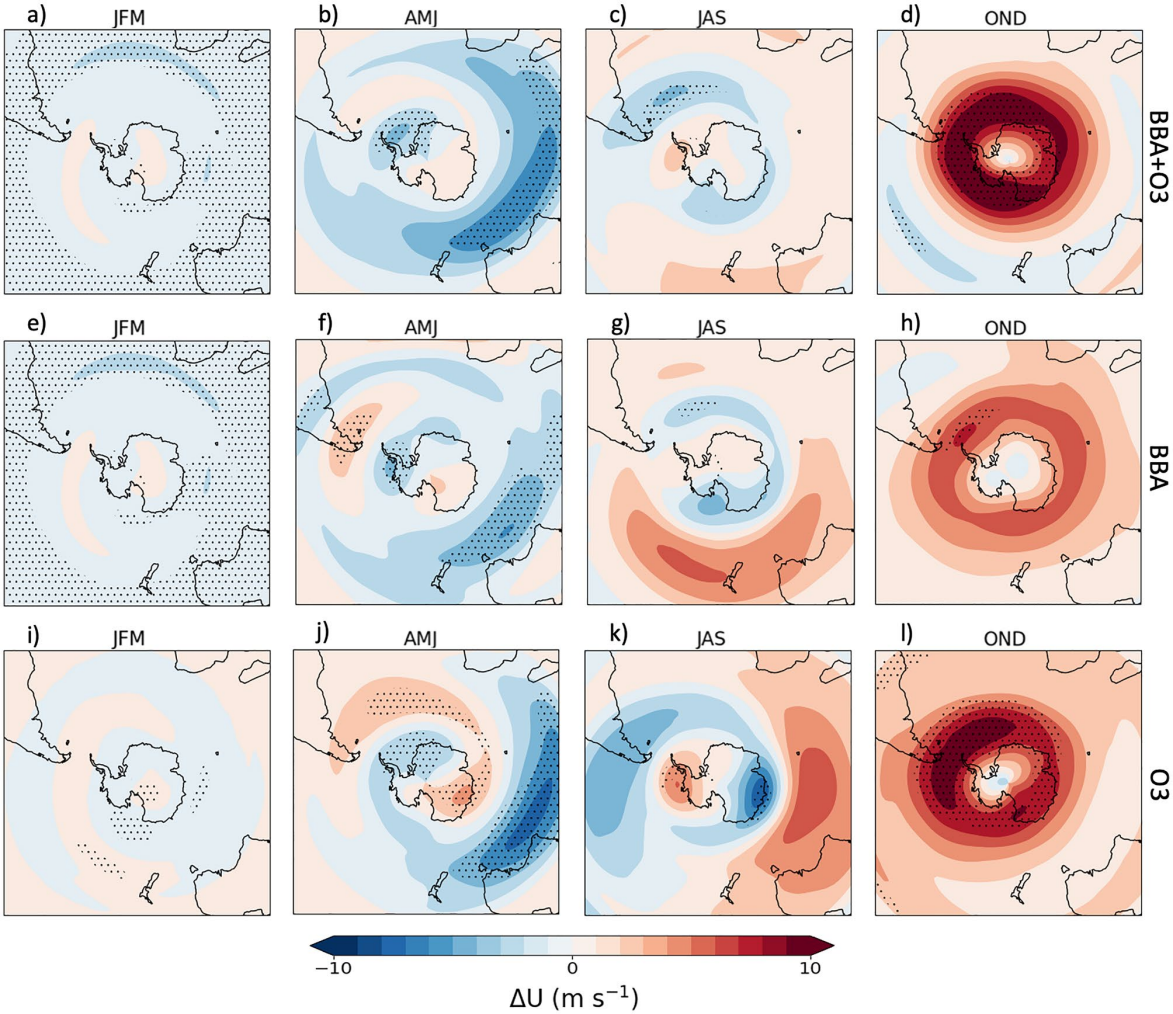
Three-monthly mean modelled perturbations the zonal mean wind (m s^-1^) at 10 hPa for 2020, over the Antarctic region, indicating the impact on the polar vortex, due to the smoke aerosol (**e–h**), the ozone anomaly (**i–l**), and both the smoke aerosol and ozone (**a–d**). The stippled points show the points at which the experiments were significantly different from the control simulations at a 5% significance level (calculated by a Welch’s t-test). The map overlaid is created using the python package, cartopy.

It is suggested that ozone depletion serves to increase the strength of the polar vortex, via reduced stratospheric heating and thermal wind balance, providing a positive feedback that appears to delay the breakdown of the polar vortex^31^. Previous studies^27,32^, note that a stronger, colder and more persistent polar vortex occurred in 2020. To further investigate impacts on the polar vortex, we plot the perturbation in the U-component of wind at 10 hPa in Fig. 8.

In January-March there is a general reduction in mid-latitude 10 hPa westerlies in response to the BBA-induced mid-latitude heating which reduces the equator-pole temperature gradient and the strength of the stratospheric circulation (Fig. 8a,e). For April-September there is some impact to the zonal winds, but no clear/coherent impact to the polar vortex can be seen until October-December; this seasonally dependant impact on stratospheric winds is consistent with previous studies (e.g.^31^). Figures 8d,l show a statistically significant strengthening of the polar vortex, by up to 10 m s^-1^, resulting from the smoke-induced ozone anomaly, which indicates that, in response to the BBA-induced ozone depletion, UKESM1 suggest that the 2020 polar vortex would be significantly stronger and colder than average. The acceleration and delayed breakdown of the polar vortex subsequently delays the mixing of ozone-rich air into the vortex interior resulting in a prolonged ozone hole as per observations in 2020. It appears that the residual stratospheric heating caused by the BBA equatorward of 50˚S enhances the zonal mean temperature gradient (Fig. 5d & h) which further enhances the October-December polar vortex from the BBA-induced ozone depletion, although this appears to be of lesser significance.

Recent studies (e.g.,^54^) have considered the impact of absorbing aerosol in the stratosphere on stratospheric water vapour content. We investigate the impact of BBA on the stratospheric water vapour content (Supplemental Fig. S5), finding a statistically significant increase in lower stratospheric water vapour within the model owing to reduction in the efficiency of the cold-trap at the tropopause.

## Discussion

While the UKESM1 model is in remarkable agreement with the observations when the BBA is included within the model for the first four to five months of 2020, it does not adequately replicate the intense cooling that peaked in October 2020 (Fig. 6; Supplemental, S5), even when the O3 anomaly is included. Reasons for this are currently unclear and we believe this should be the focus of future work. Nevertheless, the similarity between the observed warm LST perturbations and our physical modelling combining satellite retrievals and a state-of-the-art climate model leads us to conclude that the highly absorbing biomass burning aerosols from the 2019–20 South East Australian wildfires caused the highest global mean LST anomaly since that caused by the 1991 eruption of Pinatubo. As in earlier work^27^, we conclude that the smoke-induced ozone loss and resulting cooling of LSTs, particularly over the Antarctic, resulted in an acceleration and subsequent delayed breakdown of the polar vortex. This, in turn, contributed to the prolonged ozone hole that was observed in 2020. It is likely that future climate change will increase the frequency and intensity of wildfire events (e.g.^55,56^) increasing the probability of more frequent stratospheric BBA events in the future.

## Methods

This study uses native night-time CALIOP and OMPS-LP retrievals to derive weekly average aerosol extinction vertical profiles and SAOD, for the period 29 December 2019 to 31 December 2020, at 10-degree latitude bands from 20 to 70˚S. This latitude range is selected to remove the impacts of contamination of the BBA SAOD by residual impacts from the eruption of Ulawun in Papua New Guinea in June 2019, which impacted latitudes north of 20˚S and to remove data-sparse areas south of 70˚S. Most BBA smoke produced by the ANY wildfires was observed to be contained within this latitude range^18^. The long-term ‘background’ aerosol extinction vertical profiles and SAOD is calculated for the same date range, for both CALIOP and OMPS-LP, as weekly averages and at 10-degree latitude intervals, using data from June 2012 to December 2018. The long-term average excludes retrievals from 14 February to 31 December 2014 due to the eruption of Mt Kelut in Indonesia^57^. Retrievals from 22 April 2015 to 11 July 2016 are also excluded as they appear anomalously high, because of the injection of SO_2_ into the UTLS from explosive volcanic eruptions originating from South America (Calbuco) and Indonesia (e.g., Soputan, and an unidentified volcano on Gorontalo Island) and unusually active emissions from Ambrym and other volcanoes in Vanuatu^58^. The perturbations in aerosol extinction coefficient and SAOD over the period January-December 2020 that are predominantly due to the ANY wildfires are calculated by subtracting the long-term background retrievals from the 2020 retrievals. The uncertainty in the aerosol retrievals is discussed in the supplemental material, section S3.

### M1. CALIPSO/CALIOP aerosol data and quality assurance

This study uses measurements of the aerosol extinction coefficient (km^−1^) vertical profiles at 532 nm, taken from the Version 4.20 (V4) CALIOP Level 2 data product, aboard the CALIPSO satellite. CALIOP is a dual-wavelength (532 nm and 1064 nm) polarization-sensitive lidar which provides high-resolution vertical profiles of aerosols and clouds. Although lidar retrievals are active rather than passive sensors, they are susceptible to solar contamination resulting in poorer performance during the daytime^59^. Additionally, minimum detection thresholds for the aerosol extinction coefficient are estimated as 0.012 km^−1^ at night and 0.067 km^−1^ during the day^37^. Therefore, only aerosol extinction coefficient profile retrievals taken from CALIPSO night-time overpasses are used in this study.

Prior to analysis, quality control procedures, similar to those implemented in previous studies (e.g.^60^), are applied to the data. For a given vertical profile the extinction coefficient value is considered quality-assured if, for the corresponding entries (i) Extinction_QC_Flag_532 is equal to 0, 1, 2, 16, or 18, (ii) Extinction_Coefficient_Uncertainty_532 < 10, (iii) Minimum_Laser_Energy ≥ 0.08 Joules. Extinction_QC_Flag_532 reports on the type of retrieval used when solving the extinction, aerosol extinction retrievals with a corresponding Extinction_QC_Flag_532 set to the above values are deemed to be stable and the solution falls within a range of acceptable values for the aerosol optical depth (AOD) and the magnitudes of extinction coefficient^60^. Extinction_Coefficient_Uncertainty values greater than 10 represent increasingly unrepresentative aerosol extinction coefficient values^60^. Since September 2016, CALIOP has been experiencing low-energy laser shots, which primarily influence the quality of profile retrievals over the South Atlantic Anomaly (SAA) region. Hence, any profiles affected by 532 nm laser energies less than 0.08 Joules are excluded from this study. In addition, entries with a fill value of -333 are removed and entries with a fill value of -9999 are set to zero.

### M2. OMPS-LP aerosol data and quality assurance

In addition to the CALIOP aerosol extinction data, we utilise retrievals of the vertical profile of the aerosol extinction coefficient (km^−1^), taken from OMPS-LP Level 2 aerosol extinction coefficient daily product. The OMPS-LP views the Earth’s limb, looking back along the orbital track, and measures the limb scattered radiation in the UV, visible, and near-infrared wavelengths to retrieve ozone density and aerosol extinction coefficient profiles^61^. This study uses non-cloud-filtered aerosol extinction coefficient profile retrievals, measured at 869 nm along the centre slit (aligned with the orbital track) of the OMPS-LP. 869 nm is chosen rather than wavelengths closer to the peak of the solar spectrum (e.g., 510 nm), because there are clear performance issues at shorter wavelengths^62^. The non-cloud-filtered retrievals are used since the stratospheric aerosol produced by the ANY fires should be above any tropospheric cloud, and to avoid any misclassification of the optically thick aerosol plume as cloud.

**Figure 9:**
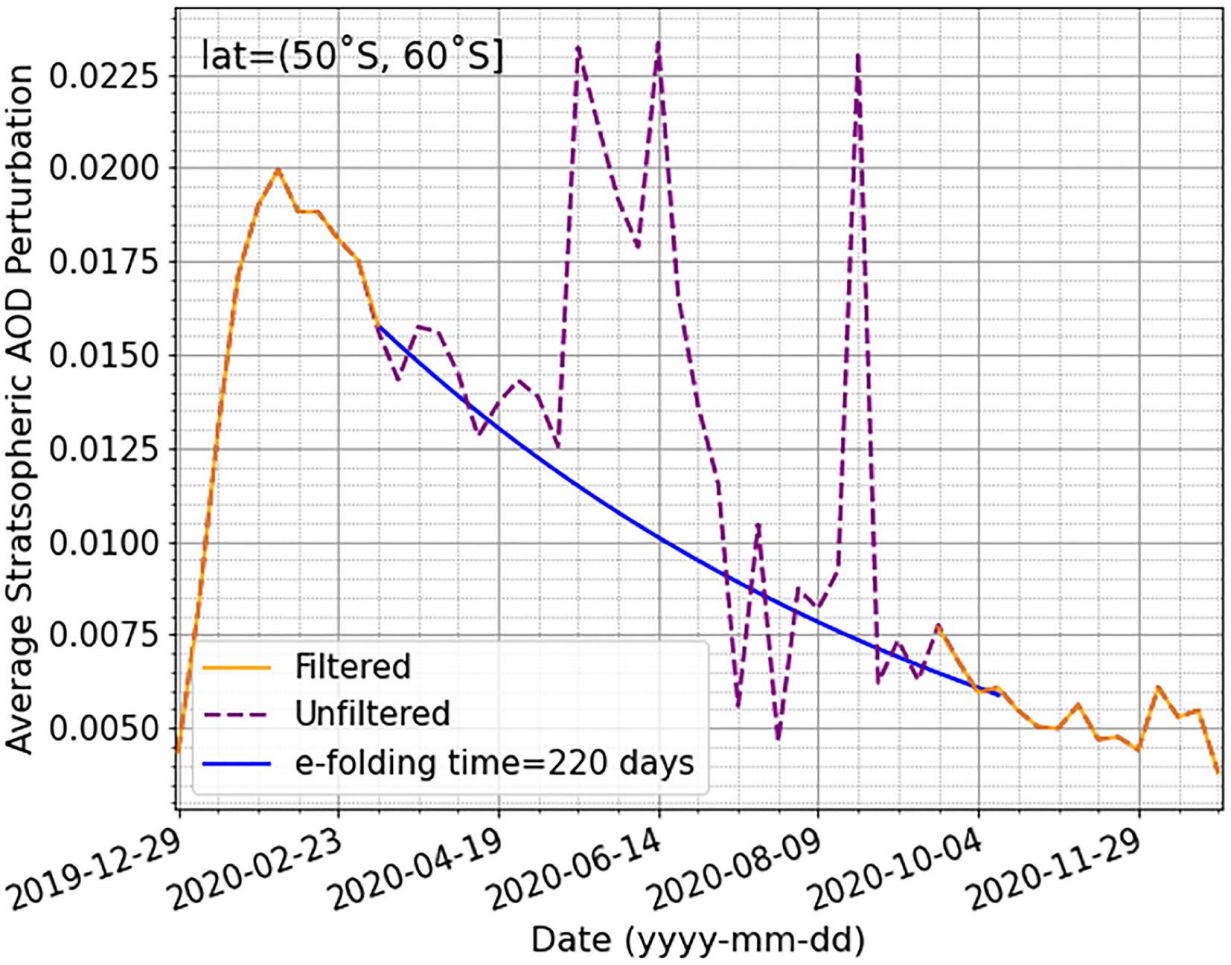
Weekly average perturbation in the SAOD (532 nm) following the ANY wildfires, over latitudes 50 to 60˚S, as observed by OMPS-LP including retrievals with a single scattering angle greater than 145˚ (purple dashed line) and with these retrievals removed (orange line). All retrievals are scaled from 869 to 532 nm assuming an appropriate Ångstrom exponent. The blue line shows a constant rate of decay of the SAOD, interpolating the filtered data, calculated from an e-folding time of 220 days.

As with the CALIOP data, quality control procedures are applied to the OMPS-LP data prior to analysis. The vertical profile of the aerosol extinction coefficient is considered quality assured if, for the corresponding entries (i) ResidualFlag = 0, (ii) SingleScatteringAngle ≤ 145˚, (iii) SwathLevelQualityFlags (a 16-bit integer) with bits 0, 1 and 7 = 0. A ResidualFlag of non-zero indicates that when retrieving the aerosol extinction profile, the cumulative residual error in the retrieval exceeds a threshold value^61^. Caution is advised when using LP extinction data with a scattering angle greater than 145˚ for wavelengths shorter than 675 nm^61^. However, Fig. 9 shows that the average SAOD at 869 nm becomes unreasonable when including the extinction retrievals with a scattering angle > 145˚, thus these are screened out. To fill in the gaps in the dataset that result from screening out these retrievals (below 30˚S, mainly during the southern hemispheric winter), a constant rate of decay of the SAOD within each latitude band is assumed (Fig. 9). The rate of decay assumed (k = 1/t) is given by the e-folding time (t), calculated as the time it takes for the SAOD to reduce by a factor of 1/e. Bits 0 and 1 of SwathLevelQualityFlags refer to the probability that the retrieval is affected by the SAA. If both bits are set to zero, this indicates that the estimated SAA effects are < 5% of the nominal maximum value at the satellite location^61^. Bit 7 of SwathLevelQualityFlags is the ‘Non-Nominal Attitude’ value which indicates changes to the spacecraft orientation, if this flag is set increased extinction profile noise is possible^61^. In addition, entries with a fill-value set to -999 are removed from the dataset.

Both CALIOP and OMPS-LP have known limitations in their aerosol extinction retrievals. As mentioned above, CALIOP has an estimated minimum detection threshold of 0.012 km^−1^ at night and a vertical resolution of 180 m in the stratosphere, whereas OMPS-LP can reliably detect aerosol extinction coefficient values down to a minimum value of 1 × 10^−5^ km^−1^^61^ and has a vertical resolution of 1 km. OMPS-LP suffers from loss of sensitivity of short wavelength radiances to aerosols, which is most pronounced below ~ 17 km and in the southern hemisphere^61^. In addition, the OMPS-LP extinction retrieval becomes unreliable in the presence of optically thick aerosol plumes^61^. The OMPS-LP retrieval issues are significant during the early stages of our study, during the fire emissions, as the aerosol was injected into the stratosphere at altitudes of ~ 16 km^17^. However, once the self-lofting of the aerosol occurs, and it ‘spreads out’ over the southern hemisphere, the increased SAOD is expected to be more detectable by OMPS-LP^18^, but becomes undetectable by CALIOP due to its minimum detection threshold being considerably higher than OMPS-LP.

### M3. Development of a composite stratospheric aerosol dataset

Due to the limitations above, the CALIOP and OMPS-LP aerosol extinction coefficient datasets are combined into one interpolated dataset; this starts as solely CALIOP data in the initial weeks of the study, then shifts over to the OMPS-LP data. The extinction vertical profiles and SAOD values for each 10-degree latitude band are linearly interpolated in time only during the ‘crossover’ period. Linear interpolation is chosen to avoid the use of excessively complicated calculations to transition between the two datasets, whilst still producing a physically reasonable dataset. Prior to the interpolation, the CALIOP vertical profiles are converted from a 180 m vertical grid to a 1 km vertical grid, by calculating the weighted average aerosol extinction coefficient for each interval, to match the vertical resolution of the OMPS-LP profiles. This conversion for the CALIOP data preserves the observed SAOD. Additionally, the OMPS-LP aerosol extinction coefficient and SAOD retrievals are scaled from 869 to 532 nm, to ensure the interpolated values are at a single wavelength. We additionally calculate the average SAOD perturbation over the whole latitude range (20–70˚S). When doing so, each SAOD measurement is weighted by the cosine of its corresponding latitude to ensure the average SAOD is area weighted.

### M4. OMPS-LP ozone data and quality assurance

Monthly average ozone density vertical profiles taken from OMPS-LP Level 2 O3 daily product and observed in the visible wavelengths, zonally averaged on a 1˚ latitude grid (between 0˚ to 90˚S), for altitudes between 12.5 and 37.5 km, are also utilised. The OMPS-LP ozone profiles observed in UV are not used in this study because the corresponding retrieval altitudes 29.5–52.5 km lie outside of the altitude range considered here. As with the aerosol retrievals, quality control procedures are applied to the ozone retrievals prior to analysis and inclusion in the model (as suggested in^63^). The ozone density profile is considered quality-assured if, for the corresponding entries (i) O3VisQuality = 1, (ii) Q_Vis = 0, (iii) eventNumber < 170, iv) SwathLevelQualityFlags (a coded flag containing five values in the form abcde) with a = 0 and e = 0. O3VisQuality is set to 1 when the retrieval was successful, and a Q_Vis value of zero indicates that the magnitude of the root sum square radiance residual from the retrieval is less than 0.05. The eventNumber represents the position of each event during each orbit, it is advised that retrievals with eventNumber ≥ 170 are likely to be affected by solar intrusion^63^, thus are removed for this study. Similar to the OMPS-LP aerosol retrievals, non-zero values for parts a and e of SwathLevelQualityFlags indicate a probability > 5% that the retrieval is affected by the SAA and increased noise due to ‘Non-Nominal Attitude’ respectively, therefore these retrievals are also removed from the dataset.

As with the aerosol profiles, the long-term ‘background’ ozone density vertical profiles are calculated using data from 2012 to 2019, excluding 2015 due to the ozone impacts of the eruption of Calbuco ^64^. The background ozone densities are then subtracted from the 2020 retrievals to produce the observed ozone anomalies in 2020 that are used in this study. OMPS-LP cannot make retrievals during the polar night and therefore observations at high latitudes in the southern hemisphere are flagged as missing data. Here, we approximate the perturbation to the ozone using linear interpolation in time for latitudes < 70˚S, for latitudes > 70˚S the ozone vertical profile for the degree below is assumed. To establish whether the interpolation procedure is successful, we present the ozone perturbation derived from our interpolated OMPS-LP data across the region 60–90˚S in Table 1. The results suggest a negative ozone anomaly of around 6DU for the period May-Aug, but reaching 16DU for September and 22DU for October. These results are in reasonable agreement with MLS ozone data^27^, suggesting that, although not perfect, the interpolation procedure appears reasonable.

**Table 1:** The monthly average OMPS-LP ozone anomaly (DU) for the lower stratosphere (~ 13–22 km) for latitudes 60–90˚S, after applying the interpolation procedure to approximate the missing data during the SH winter.

Month	Jan	Feb	Mar	Apr	May	Jun	Jul	Aug	Sep	Oct	Nov	Dec
Ozone anomalyDU	2.2	0.2	− 0.7	− 4.5	− 6.0	− 6.0	− 6.0	− 3.6	− 16.2	− 22.5	− 34.8	− 25.9

### M5. The UKESM1 climate model and simulations

We used the atmosphere-only version^65,66^ of the United Kingdom Earth System Model version 1 (UKESM1;^67^) which includes 85 model levels from the surface to 85 km at a resolution of 192 × 144 longitude-latitude grid-boxes and includes interactive tropospheric and stratospheric chemistry and aerosols^68–70^. For the period 1850–2014, the control simulation is a coupled run including the historical period^70^. Other studies^9,70^ suggest that the UKESM1 model performance is reasonable for both ozone and stratospheric temperature trends. The control simulation then transitions to a further 15-year period (2015–2029) where greenhouse gas concentrations, aerosol-chemistry emissions, and sea surface temperatures (SST) were maintained at 2014 levels to create a sample “present-day” climate using background stratospheric aerosol climatologies. Thus, each year of the control after 2015 had the same forcing but different initial conditions and meteorological flow patterns. Yearly sections of the control from 2016 to 2025 (CNTL) were then used to form the basis of a 10-member ensemble with and without the COMP and with and without the observed aerosol and stratospheric ozone perturbations. All other model settings, including the SSTs were identical. Perturbation simulations ran for a full year from January—December and each followed a different meteorological evolution according to the initial conditions provided. The rationale for such an approach is that it samples different phases within the cycles of stratospheric natural variability such as the Quasi Biennial Oscillation, which are represented well by the UKESM1 model (e.g. ^71^).

The COMP aerosol properties were included in the perturbation simulations using the model’s Easy Aerosol system. In this approach, aerosol optical properties are specified from an external data source and provided directly to the model’s radiation scheme. The Easy Aerosol method was used for UKESM1 in simulating stratospheric aerosol during the Coupled Model Intercomparison Project 6 (CMIP6)^66^. In the perturbation runs the scattering and absorption associated with the BBA were added on top of the background (historical mean) volcanic aerosol used in the control simulation. The BBA optical properties were set to match the aerosol extinction in the COMP data (described above) and were included as weekly, height resolved zonal-mean values, interpolated onto the UKESM1 grid. The wavelength dependence of the extinction, the single scattering albedo and the asymmetry parameters were derived from Mie scattering theory using size distributions retrieved from the AERONET station at Punta Arenas (Chile). The AERONET data was taken as the mean from all available retrievals during 26–30 January 2020 when the smoke originating from Australia was observed in the stratosphere above the Southern tip of South America and was dominating the AOD (0.24–0.28), relative to the background mean (0.056) taken from 2019 to 2021. The composition of the aerosol was assumed to be a homogeneous internal mixture with 5.4% black carbon (BC) and 94.6% organic carbon (OC) with mass densities of 1.9 g cm^-3^ and 1.35 g cm^-3^, respectively, based on aircraft measurements^72^. An updated BC refractive index of 1.85 + 0.71i at 550 nm was assumed^73^ and, when combined with an assumed non-absorbing OC refractive index of 1.50 + 0i and a volume-weighted averaging approach, yields an effective refractive index of 1.54–0.027i. These above assumptions lead to a single scattering albedo of 0.86 at 550 nm. This is consistent with recent observations of aged smoke in the troposphere^41^. The single scattering albedo varies across the solar spectrum, with values of 0.822, 0.849, 0.859, 0.858 and 0.855 at wavelengths 300 nm, 450 nm, 550 nm, 670 nm and 870 nm respectively; the lower values at short wavelengths being commensurate with absorption by organics^74^. In the troposphere, observations show the chemical composition, mixing state, and morphology of biomass burning aerosol to be highly variable as they depend on the combustion source and moisture content and the degree of smouldering and flaming combustion and hence the optical properties of biomass burning aerosols are extremely variable. In the upper troposphere and stratosphere, the uncertainty in the refractive indices, particularly in the imaginary part of the refractive index and single scattering albedo will be even larger owing to complexities associated with oxidation, bleaching, evaporation of volatile organic and inorganic components, the collapse of carbon chain structures, etc. (e.g.^46^). There is clear evidence emerging that stratospheric BBA smoke is more absorbing than tropospheric BBA smoke, for example, a single scattering albedo at 532 nm of 0.8–0.9 and 0.80 for aged Canadian boreal fires^44,45^. Additionally, lidar observations of SE Australian smoke that reached South America during the episode that we study here^23^ displayed considerably higher lidar ratios (extinction/backscatter) which are indicative of more highly absorbing aerosol than their Canadian boreal BBA counterparts.

Stratospheric ozone perturbations are treated in a similar way to stratospheric aerosol perturbations. The perturbations derived from OMPS-LP (Fig. 4) are added to the background model ozone.

We perform three separate UKESM1 model simulations each comprised of 10 pairs of simulations. Each pair includes a control simulating present-day climate (CNTL) and one of three parallel simulations starting from the same initial conditions but including either the aerosol perturbation (BBA), the ozone perturbation (O3), or both the aerosol and ozone perturbations (BBA + O3). Each pair runs for 12 months, using a unique set of initial conditions to create an ensemble of results.

### M6. Stratospheric temperature anomaly data

We consider observations of the LST anomaly in the lower stratosphere, based on measurements made by Microwave Sounding Units (MSU) channel 4 and Advanced Microwave Sounding Units (AMSU) channel 9 on polar-orbiting satellites. We use the TLS (Temperature Lower Stratosphere) single-channel data set of zonally averaged temperature anomalies, produced by Remote Sensing Systems (RSS), which is sensitive to temperatures between altitudes of 12 km to 27 km. This dataset includes data from 13 different satellites, which are inter-calibrated before being merged^75^. The mean temperatures (T_b_) used in this data set are given by: -

1 $$T_{b} = W_{s} T\left( 0 \right) + \mathop \int \limits_{0}^{TOA} W\left( z \right)T\left( z \right) dz$$

where *T*(*z*) is the atmospheric temperature at altitude *z*, *W*(*z*) is a weighting function and *Ws* is the surface weight^75^. The RSS data set provides a continuous record of monthly temperature anomalies, from 1979 to the present day, averaged over several latitude bands (between 83˚S and 83˚N). The anomalies are computed by subtracting the mean monthly value, determined by averaging over a 1979–2019 reference period, from the average temperature for each month.

## Supplementary Information

Supplementary Information.

## Data Availability

Observational and model data is available from the Centre for Environmental Data Analysis archive (CEDA) catalogue (https://archive.ceda.ac.uk/), project reference: NE/S00212X/1. MSU/AMSU data are produced by Remote Sensing Systems and sponsored by NASA (data are available at www.remss.com). Assistance in analyzing the data is available by contacting the corresponding author.
