# Supplementary material for: Australian wildfires cause the largest stratospheric warming since Pinatubo and extends the lifetime of the Antarctic ozone hole
Source: Sci Rep. 2022 Aug 25;12:12665. doi: 10.1038/s41598-022-15794-3 (PMC9411169; doi:10.1038/s41598-022-15794-3)
Supplement: Supplementary file 1 — Supplementary Information. [file 41598_2022_15794_MOESM1_ESM.pdf]

SUPPLEMENTAL MATERIAL

Australian Wildfires cause the largest stratospheric warming since Pinatubo and extends the lifetime of the Antarctic ozone hole.

Lilly Damany-Pearce, Ben Johnson, Alice Wells, Martin Osborne, James Allan, Claire Belcher, Andy Jones, Jim Haywood

S1) Qualitative assessment of the early evolution of the biomass burning aerosol (BBA) smoke plume.

In addition to its limb-sounding capabilities, OMPS on the SUOMI satellite carries a nadir mapping (OMPS-NM) spectrometer operating in the 300–380 nm wavelength range at a resolution of 1 nm (e.g., Yang et al., 2013). These wavelengths are proven in making global semi-quantitative measurements of absorbing aerosol, owing to the spectral invariance of the surface at such wavelengths and the lack of significant influence of clouds relative to absorbing aerosols on detection algorithms (e.g., Torres et al., 2007). Retrieval techniques are well proven for detection of both mineral dust and biomass burning smoke aerosols for predecessor instruments such as the Total Ozone Mapping Spectrometer (TOMS, Christopher et al., 2008) and the Ozone Mapping Instrument (OMI, Torres et al., 2007), but are only semi-quantitative owing to the dependence of the retrievals on both the absorption of the aerosol and the altitude where the aerosol resides (e.g., Christopher et al., 2011). The current OMPS-NM sensor has been used to track the evolution of stratospheric BBA plumes (e.g., Torres et al., 2020, Peterson et al., 2021, Christian et al., 2019).

The OMPS-NM retrievals do not provide aerosol optical depths, but the so-called “Aerosol Index (AI)”. The AI uses two ultraviolet (UV) wavelengths and separates the impacts caused by absorbing aerosols from that of other effects, including molecular Rayleigh scattering, surface reflection, gaseous absorption and aerosol and cloud scattering (Torres et al., 1998; de Graaf et al., 2005). Figure S1 shows the evolution of the OMPS-NM AI over the first month of the event.

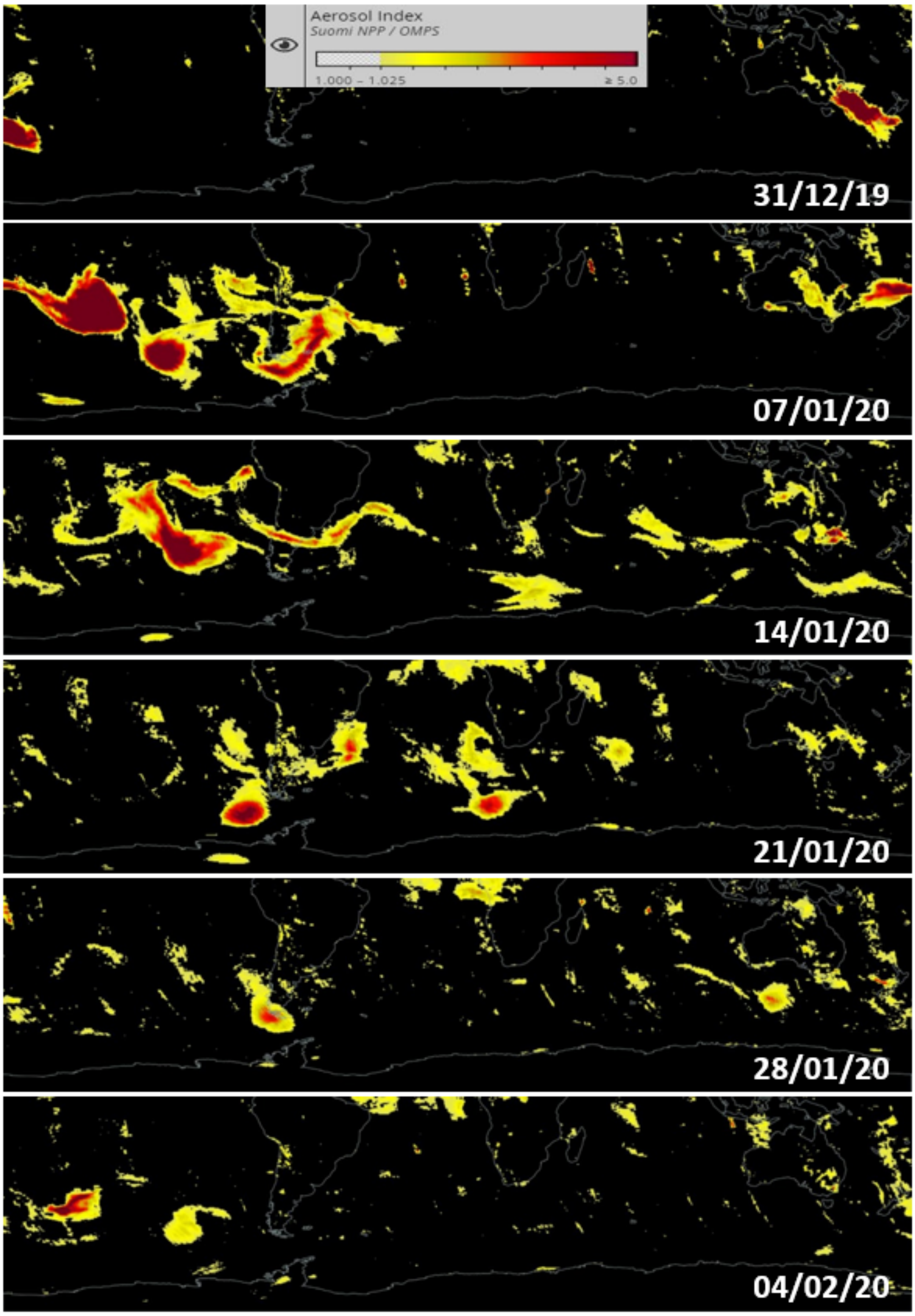

Figure S1. Showing the Aerosol Index derived from the OMPS-NM sensor on-board the SUOMI satellite over the first month of the event. The red and yellow areas show high concentrations of absorbing aerosols. Aerosol Indices of less than 1 are masked from the plots to emphasize the progression of the biomass burning smoke during the time period. Images created using NASA Worldview (https://worldview.earthdata.nasa.gov/).

S2) Diagnosing the Heating Rate caused by the BBA smoke perturbation

Before assessing the temperature perturbation caused by the BBA smoke, we assess the magnitude of the induced heating rates (Figure S2) that are provided as an output in the UKESM1 climate model. It is clear that the maximum heating rate perturbations correspond to the BBA smoke and reach a maximum in January of more than 0.2 K/day. Heating rates are generally displaced to higher altitudes than the maximum in the aerosol extinction owing to the exponential dependence of the attenuation of sunlight per unit aerosol optical depth, and also displaced to equatorial latitudes during autumn/wintertime (e.g., May/June) owing to reduced insolation at high latitudes.

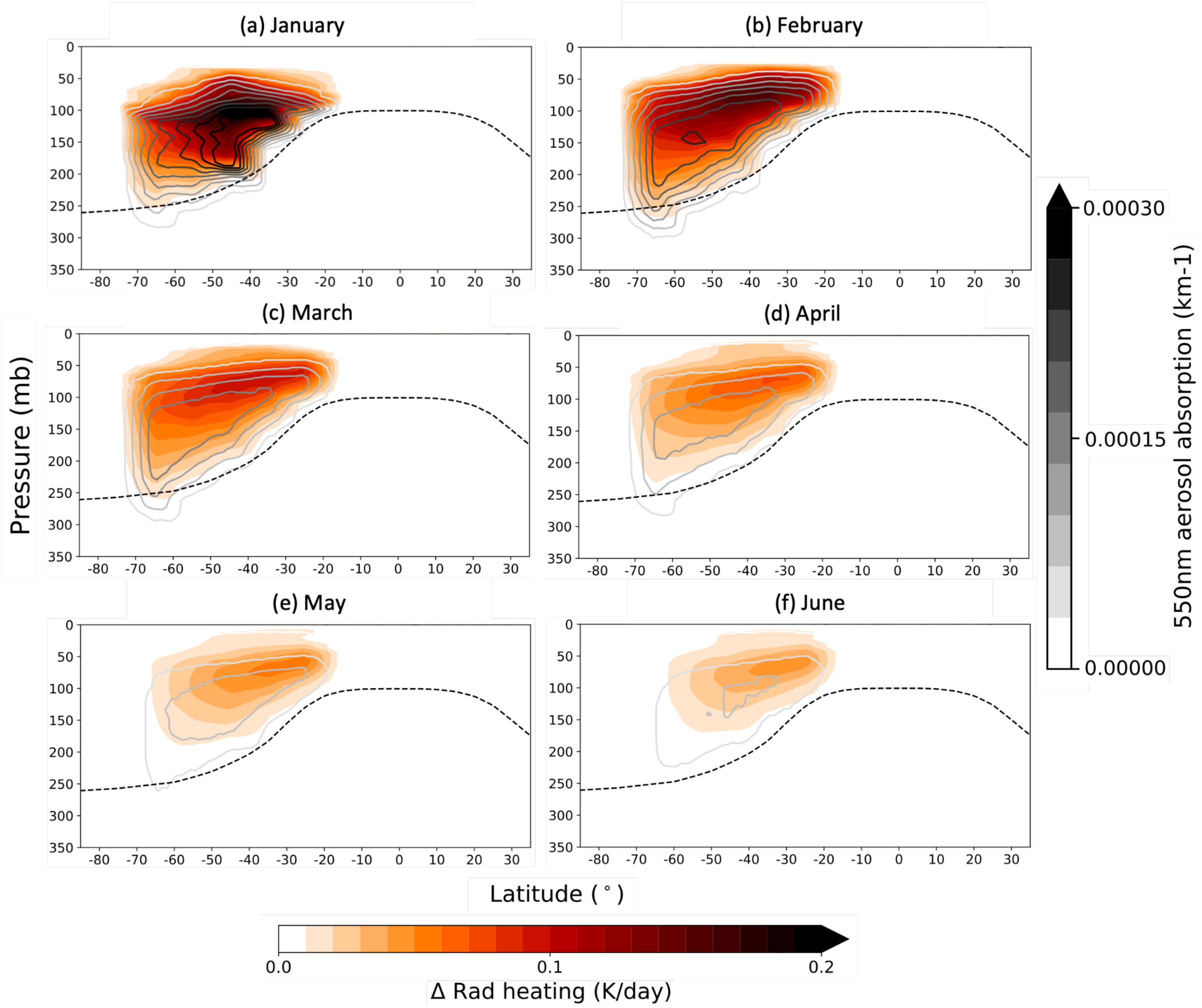

Figure S2. Monthly mean heating rate perturbation (K/day) caused by BBA smoke diagnosed over the months January-June 2020. The average monthly aerosol absorption (km^{-1}) is overlaid. The black dashed line shows the average tropopause height from the UKESM1 model climatology

S3) Assessment of the uncertainty in the observations

Both CALIOP and OMPS-LP provide an extinction uncertainty product for each aerosol extinction retrieval, we use these to assess the uncertainty in our COMP aerosol dataset. The same data screening methods that are applied to the extinction profiles, detailed in methods M1 and M2, are applied to the uncertainty data, then the monthly averages are calculated. Figure S3 shows that the uncertainty in the OMPS-LP retrievals stays at around 2% of the extinction coefficient within the location corresponding to the most intense BBA plume, during February when the SAOD perturbation was the greatest (Figure 2). However, Figure S3 shows the CALIOP retrievals have a much larger relative uncertainty, reaching up to 70% of the extinction coefficient in the middle of the aerosol plume. Although the when comparing to the OMPS-LP uncertainty the CALIOP uncertainty is large, we allow it for this study, as sufficient data screening methods have been applied (see methods M1) and the CALIOP data is only used in the COMP dataset for the first 3 weeks (Figure 2). The uncertainty in the OMPS-LP extinction retrievals remains below 10%, within the main aerosol plume, for most of 2020, until the plume starts to dissipate and the uncertainty increases to 15% in some areas from September.

OMPS-LP does not provide an uncertainty product for the ozone retrievals in the same way it does for the aerosol extinction coefficient, however the data screening methods described in Methods, M4, quality assure the ozone data, keeping the uncertainty to a minimum. Additionally, DeLand et al., (2017), provide a detailed analysis of the sources and estimated quantities of the uncertainty within the dataset, including comparison to MLS data.

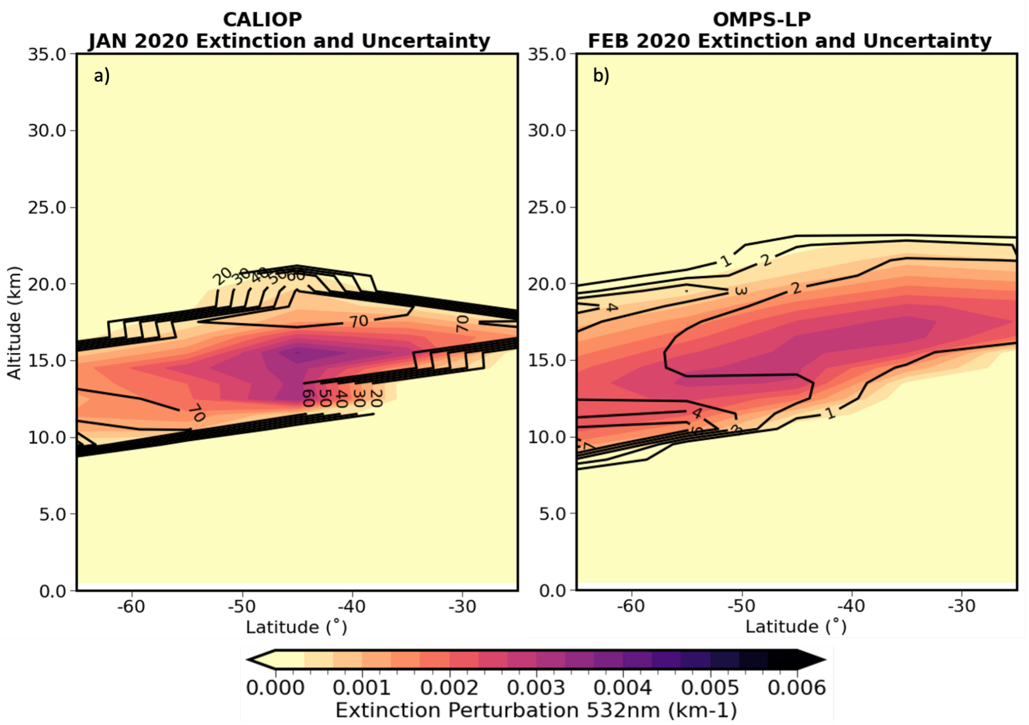

Figure S3. The monthly average CALIOP (a) and OMPS-LP (b) retrieved aerosol extinction perturbation for January and February respectively, when the stratospheric smoke plume was most intense. The OMPS-LP extinction has been scaled to 532nm using an appropriate Ångstrom exponent so it is comparable to the CALIOP data. The overlaid contour lines show the uncertainty in the retrievals as a percentage of the retrieved aerosol extinction coefficient.

S4) Assessment of the absorption from stratospheric BBA smoke relative to sulfate aerosol

The impact of the relatively minor increase in stratospheric AOD on LSTs caused by BBA is clarified here. We derive aerosol scattering and absorption parameters (k_{sca} and k_{abs}) assuming Mie scattering theory for both sulfate and BBA smoke. For stratospheric sulfate, we assume a simple mono-modal gamma distribution as per WCP (1986). For BBA, we use a size distribution retrieved by the AERONET station at Punta Arenas (South America), averaged over the period 26 - 30th January 2020 when the stratospheric smoke layer was overhead and dominated the AOD. The refractive indices for the BBA were based on Peers et al., (2019) (see methods) and yield a single scattering albedo of 0.86 at 550nm. We calculate the optical properties across the 0.2-10μm wavelength range and weighting the derived optical properties by the incident top of the atmosphere solar flux to provide appropriate solar spectral irradiance weighted optical properties. The resulting specific scattering (k_{sca}) and specific absorption (k_{abs}) coefficients, the single scattering albedo (SSA) and the co-albedo (1-SSA) are presented in Table S1. Densities of 1.769g cm^{-3} and 1.35g cm^{-3} are assumed for sulphate and for BBA respectively.

**Aerosol**	**k_{sca} (m^2g^{-1})**	**k_{abs} (m^2g^{-1})**	**SSA**	**(1-SSA)**
**Sulphate**	3.794	0.0099	0.9974	0.0026
**BBA smoke**	2.764	0.4908	0.8492	0.1508

Table S1. Broadband (0.2-10.0micron) optical parameters weighted by the top of atmosphere solar spectral irradiance.

Because the size distributions are relatively similar, peaking at sub-micron wavelengths, the specific extinction (i.e. the sum of k_{sca} and k_{abs}) is relatively similar. The co-albedo provides an estimate of the absorption of the aerosols averaged over the solar spectrum. The results suggest that BBA smoke will absorb around 50 times (i.e. 0.4908/0.0099) the amount of sunlight than sulfate on an equivalent mass column loading basis.

S5) Assessment of the impact of BBA smoke on the lower stratospheric temperatures for other regions of the globe

To make a more comprehensive assessment of the impacts of the BBA smoke on the LST, and to investigate the relative impact of the sudden stratospheric warming that occurred in September 2019 (e.g., Rao et al., 2020; Yamazaki et al., 2020), we provide analysis of observations and model simulations over the southern hemisphere (0˚-83˚S), the southern hemisphere mid-latitudes (25˚S-60˚S) and southern polar latitudes (60˚S-83˚S). These latitude bands are chosen as they are the native bands that are provided in the RSS observational records (see methods, section M4). We also provide LST data for the northern hemisphere (0˚-83˚N). All observed and modelled LST anomalies are shown in Figure S4.

The model simulations reveal statistically significant deviations (at a 95% confidence level, using a Student's t-test) from the mean temperature occur throughout the period February-May 2020, with the most significant perturbations occurring at mid-latitudes (25˚S-60˚S), where the modelled LST perturbations are the largest (Figure 3). The broad peak in LSTs that is modelled when BBA smoke is included also appears in the observations for both the southern hemisphere and for (25˚S-60˚S), again suggesting that BBA smoke is a primary explanatory driver.

In the model simulations at polar latitudes (60˚S-83˚S), there are no features that are significant at a 95% level although the temperature perturbation during February and March approaches this level of significance. At polar latitudes, there are several features in the observed LSTs that the model fails to capture. The sudden stratospheric warming (SSW) in September 2019 is clearly evident in the observations and is the largest on the RSS record. There is also the largest cooling on record that commences in September/October 2020, reaching its peak in November 2020. Our model simulations are free-running and cannot be expected to simulate the timing of the SSW event. In the observations, the cooling event subsequent in October and November 2020 peaks at around -9K and appears to be linked to maintenance of a stronger polar vortex induced by the BBA-induced ozone depletion (see main text), although our model does not reproduce as strong an impact as in the observations.

In the northern hemisphere in September 2019, there is the most significant negative LST anomaly on record which more than compensates for the impact on global LSTs from the SSW event in the southern hemisphere. Whether this event occurs by chance or is related to dynamical impacts such as the role of atmospheric blocking (e.g., Shen et al., 2020) and teleconnections between the northern and southern hemisphere stratosphere (e.g., Noguchi et al., 2020) is worthy of further research.

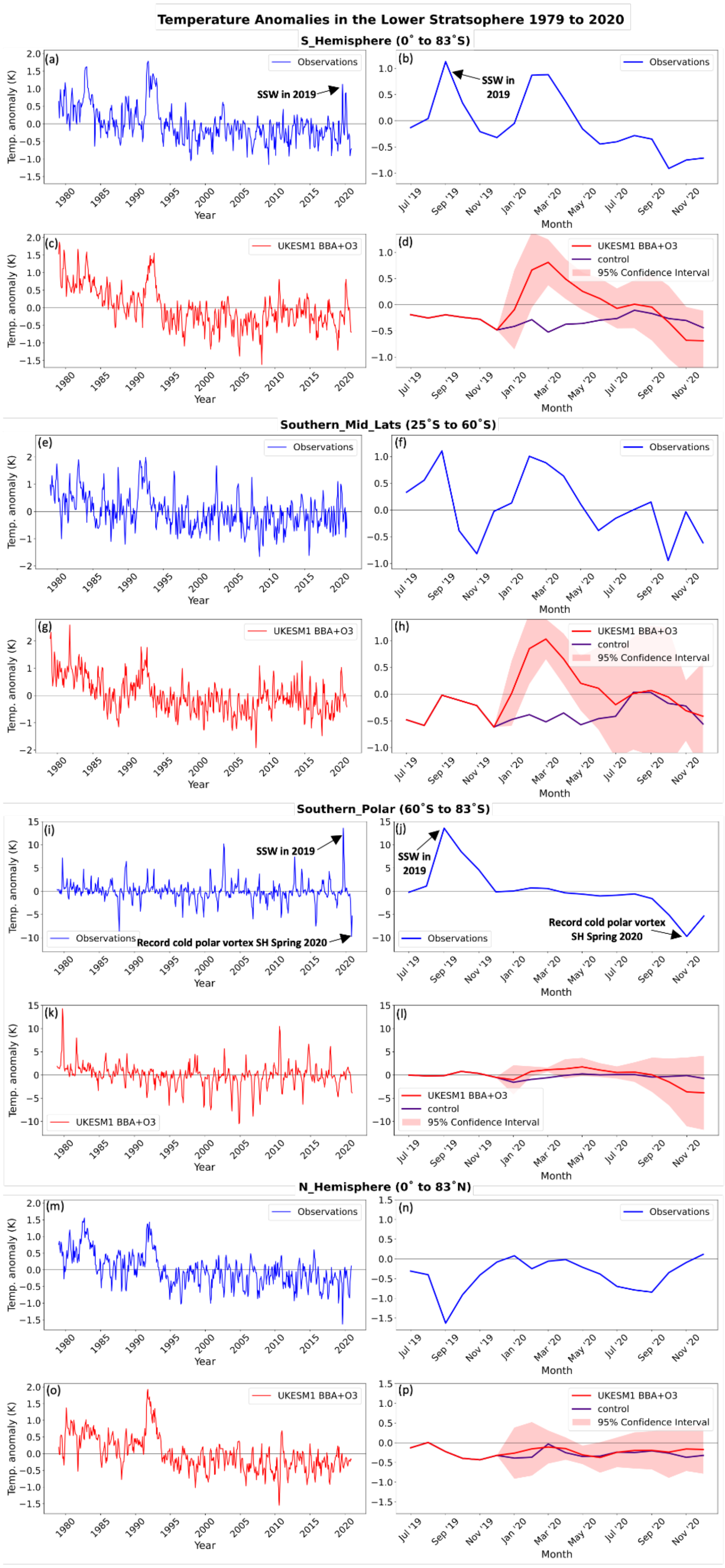

Temperature Anomalies in the Lower Stratsophere 1979 to 2020
S_Hemisphere (0˚ to 83˚S)
Southern_Mid_Lats (25˚S to 60˚S)
Southern_Polar (60˚S to 83˚S)
N_Hemisphere (0˚ to 83˚N)
(a)
(b)
(c)
(d)
(e)
(f)
(g)
(h)
(i)
(j)
(k)
(l)
(m)
(n)
(o)
(p)
Observations
UKESM1 BBA+O3
control
95% Confidence Interval
SSW in 2019
Record cold polar vortex SH Spring 2020
Temp. anomaly (K)
Year
Month

Figure S4. The monthly-mean LST anomalies, from 1979 to 2020 provided by RSS data (a,b,e,f,i,j,m,n), and the mean of 10 ensemble member UKESM1 BBA+O3 experiment runs, with the 95% confidence intervals for each month of 2020 assuming a Student's t-distribution, along with the mean of the control simulations (c,d,g,h,k,l,o,p). Temperature anomalies are calculated from a 1979-2019 reference period, and zonally averaged for each of the latitude bands. (a-d) show the temperature anomalies over the southern hemisphere (0˚ and 83˚S), (e-h) show the temperature anomalies over the southern mid-latitudes (between 25˚S and 60˚S), (i-l) show the temperature anomalies in the southern polar region (between 60˚S and 83˚S), (m-p) show the temperature anomalies in the northern hemisphere (between 0˚ and 83˚N).

S6) Assessment of the impact of BBA smoke on the stratospheric water vapour content

The troposphere-stratosphere transport across the tropopause is the predominant source of stratospheric water vapour (Holton et al., 1995). The tropopause acts as a 'cold-trap' which then limits this transport (Sherwood and Dessler, 2001) drying the air to the local saturation vapour pressure as it crosses into the stratosphere. This leads to the condensation of water vapour and the dehydration of stratospheric air. Changes in the cold point of the tropopause, resulting from the presence of absorbing aerosols in the stratosphere, could cause a decrease in the maximum relative humidity experienced in troposphere-stratosphere exchange processes. This could lead to a reduction in dehydration of air passing into the stratosphere and increase the concentrations of stratospheric water vapour. Figure S5 shows statistically significant increases in the stratospheric water vapour content, of up to 0.4ppmv, as a result of the presence of smoke aerosol in the stratosphere, over most of 2020, resulting from the Australian Black Summer fires.

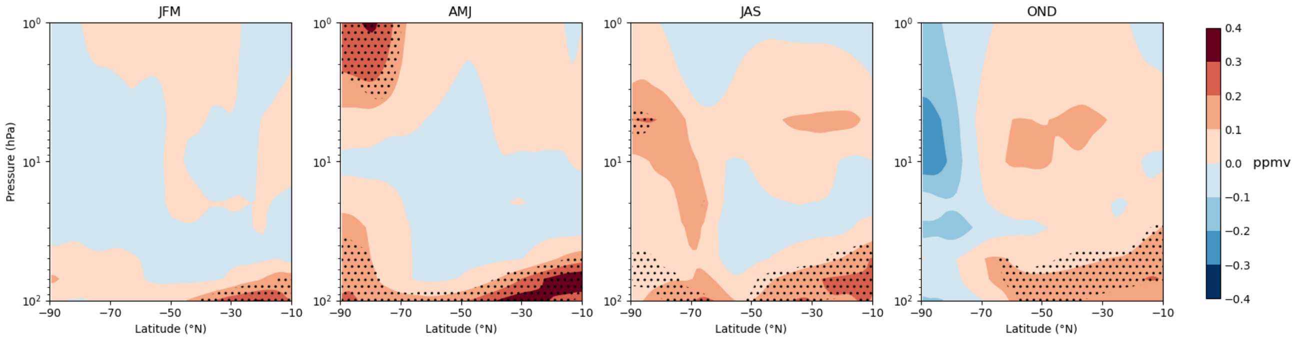

Figure S5. Three-monthly mean modelled perturbations to the stratospheric water vapour content, for altitudes 100hPa (~16km) and above over latitudes (10-90˚S), due to the BBA+O3 experiment, shown as the difference from the average of the control simulations. The stippled points show the points at which the experiments were significantly different from the control simulations at a 5% significance level (calculated by a Welch's t-test).

References: Supplemental Material

Christian, K., Wang, J., Ge, C., Peterson, D., Hyer, E., Yorks, J., McGill, M., 2019. Radiative forcing and stratospheric warming of pyrocumulonimbus smoke aerosols: First modeling results with multisensor (EPIC, CALIPSO, and CATS) views from space. Geophysical Research Letters 46, 10061–10071.

Christopher, S., P. Gupta, J.M. Haywood, G. Greed, Aerosol optical thicknesses over North Africa. Part 1: Development of a product for model validation using TOMS, MISR, and AERONET data, J. Geophys. Res., 113, D00C04, doi:10.1029/2007JD009446, 2008.

Christopher, S. A., Gupta, P., Johnson, B., Ansell, C., Brindley, H. and J.M. Haywood, (2011), Multi-sensor satellite remote sensing of dust aerosols over North Africa during GERBILS. Quart J. of the Royal Meteorological Society, 137: doi: 10.1002/qj.863, 137, 1168-1178, 2011.

De Graaf, M., Stammes, P., Torres, O. and Koelemeijer, R.B.A., 2005. Absorbing Aerosol Index: Sensitivity analysis, application to GOME and comparison with TOMS. *Journal of Geophysical Research: Atmospheres*, *110*(D1).

M. DeLand, P. K. Bhartia, P. Xu, N. Kramarova, and T. Zhu, 'OMPS Limb Profiler Ozone Product O3: Version 2.5 Data Release Notes'. Aug. 14, 2017.

Holton, J.R., Haynes, P.H., McIntyre, M.E., Douglass, A.R., Rood, R.B. and Pfister, L., 1995. Stratosphere-troposphere exchange. Reviews of geophysics, 33(4), pp.403-439.

Noguchi, S., Kuroda, Y., Kodera, K., & Watanabe, S. (2020). Robust enhancement of tropical convective activity by the 2019 Antarctic sudden stratospheric warming. Geophysical Research Letters, 47, e2020GL088743. https://doi.org/10.1029/2020GL088743.

Peers, F., Francis, P., Fox, C., Abel, S.J., Szpek, K., Cotterell, M.I., Davies, N.W., Langridge, J.M., Meyer, K.G., Platnick, S.E., 2019. Observation of absorbing aerosols above clouds over the south-east Atlantic Ocean from the geostationary satellite SEVIRI–Part 1: Method description and sensitivity. Atmospheric Chemistry and Physics 19, 9595–9611.

Peterson, D.A., Fromm, M.D., McRae, R.H., Campbell, J.R., Hyer, E.J., Taha, G., Camacho, C.P., Kablick, G.P., Schmidt, C.C., DeLand, M.T., 2021. Australia's Black Summer pyrocumulonimbus super outbreak reveals potential for increasingly extreme stratospheric smoke events. npj Climate and Atmospheric Science 4, 1–16.

Rao, J., Garfinkel, C.I., White, I.P., Schwartz, C., 2020. The Southern Hemisphere minor sudden stratospheric warming in September 2019 and its predictions in S2S models. Journal of Geophysical Research: Atmospheres 125, e2020JD032723.

Sherwood, S.C. and Dessler, A.E., 2001. A model for transport across the tropical tropopause. Journal of the Atmospheric Sciences, 58(7), pp.765-779.

Shen, X., Wang, L. and Osprey, S., 2020. The Southern Hemisphere sudden stratospheric warming of September 2019. *Science Bulletin*, *65*(21), pp.1800-1802.

Torres, O., P. K. Bhartia, J. R. Herman, Z. Ahmad, and J. Gleason (1998), Derivation of aerosol properties from satellite measurements of backscattered ultraviolet radiation: Theoretical basis, J. Geophys. Res., 103(D14), 17,099–17,110.

Torres, O., Tanskanen, A., Veihelmann, B., Ahn, C., Braak, R., Bhartia, P.K., Veefkind, P. and Levelt, P., 2007. Aerosols and surface UV products from Ozone Monitoring Instrument observations: An overview. *Journal of Geophysical Research: Atmospheres*, *112*(D24).

Torres, O., Bhartia, P.K., Taha, G., Jethva, H., Das, S., Colarco, P., Krotkov, N., Omar, A., Ahn, C., 2020. Stratospheric injection of massive smoke plume from Canadian boreal fires in 2017 as seen by DSCOVR-EPIC, CALIOP, and OMPS-LP observations. Journal of Geophysical Research: Atmospheres 125, e2020JD032579.

WCP, 1986: A preliminary cloudless standard atmosphere for radiation computation. World Climate Programme Rep. WCP-112, 54 pp

Yamazaki, Y., Matthias, V., Miyoshi, Y., Stolle, C., Siddiqui, T., Kervalishvili, G., Laštovička, J., Kozubek, M., Ward, W., Themens, D.R., 2020. September 2019 Antarctic sudden stratospheric warming: Quasi-6-day wave burst and ionospheric effects. Geophysical Research Letters 47, e2019GL086577.

Yang, K., Dickerson, R.R., Carn, S.A., Ge, C. and Wang, J., 2013. First observations of SO2 from the satellite Suomi NPP OMPS: Widespread air pollution events over China. *Geophysical Research Letters*, *40*(18), pp.4957-4962.
